# microRNAs as Early Biomarkers of Alzheimer’s Disease: A Synaptic Perspective

**DOI:** 10.3390/cells10010113

**Published:** 2021-01-09

**Authors:** Dolores Siedlecki-Wullich, Alfredo J. Miñano-Molina, José Rodríguez-Álvarez

**Affiliations:** 1 Department Bioquímica i Biologia Molecular, Institut de Neurociències, Universitat Autònoma de Barcelona, 08193 Cerdanyola del Vallès, Spain; Alfredo.minano@uab.cat (A.J.M.-M.); jose.rodriguez@uab.es (J.R.-Á.); 2 Centro de Investigación Biomédica en Red Sobre Enfermedades Neurodegenerativas (CIBERNED), 528031 Madrid, Spain; 3 Dominick P. Purpura Department of Neuroscience, Albert Einstein College of Medicine, New York, NY 10461, USA

**Keywords:** miRNAs, synaptic dysfunction, Alzheimer’s disease, biomarkers

## Abstract

Pathogenic processes underlying Alzheimer’s disease (AD) affect synaptic function from initial asymptomatic stages, long time before the onset of cognitive decline and neurodegeneration. Therefore, reliable biomarkers enabling early AD diagnosis and prognosis are needed to maximize the time window for therapeutic interventions. MicroRNAs (miRNAs) have recently emerged as promising cost-effective and non-invasive biomarkers for AD, since they can be readily detected in different biofluids, including cerebrospinal fluid (CSF) and blood. Moreover, a growing body of evidence indicates that miRNAs regulate synaptic homeostasis and plasticity processes, suggesting that they may be involved in early synaptic dysfunction during AD. Here, we review the current literature supporting a role of miRNAs during early synaptic deficits in AD, including recent studies evaluating their potential as AD biomarkers. Besides targeting genes related to Aβ and tau metabolism, several miRNAs also regulate synaptic-related proteins and transcription factors implicated in early synaptic deficits during AD. Furthermore, individual miRNAs and molecular signatures have been found to distinguish between prodromal AD and healthy controls. Overall, these studies highlight the relevance of considering synaptic-related miRNAs as potential biomarkers of early AD stages. However, further validation studies in large cohorts, including longitudinal studies, as well as implementation of standardized protocols, are needed to establish miRNA-based biomarkers as reliable diagnostic and prognostic tools.

## 1. Introduction

**Figure 1:**
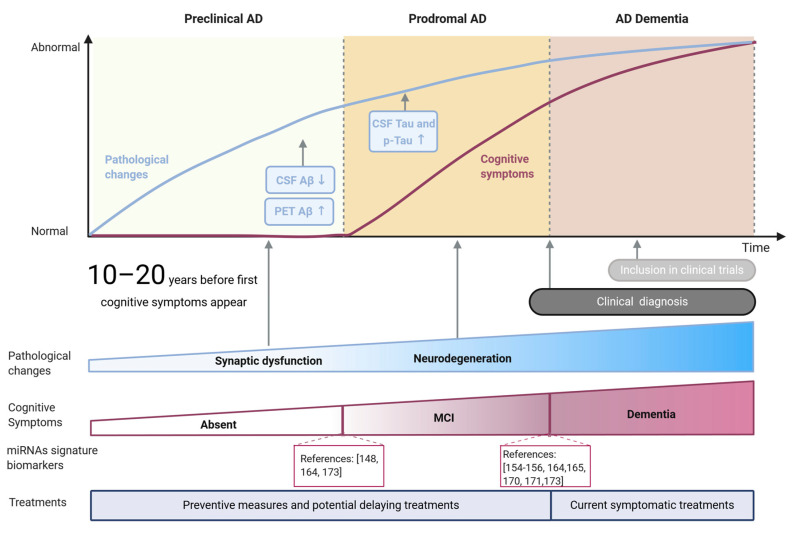
Main stages and features of Alzheimer’s disease. Pathological changes and cognitive symptoms are represented as blue and brown lines, respectively. Pathological hallmarks currently used as biomarkers (Aβ and tau) are shown in blue rectangles, while key global pathological changes are indicated with arrows. Cognitive symptoms are summarized as MCI (mild cognitive impairment) and dementia stages. miRNA-based signatures for potential diagnosis of MCI and AD stages are indicated as references.

Alzheimer’s disease (AD) is a progressive and irreversible brain disorder and the most frequent form of dementia among the elderly, reaching nearly 70% of cases [1,2]. Although the development of the disease is variable between patients, three phases can be distinguished as part of a continuous process of degeneration (Figure 1): (1) The preclinical phase, usually lasting more than 10 years, characterized by early changes in biomarkers in the absence of clinical symptoms; (2) the prodromal phase comprising the earliest symptomatic stage of the disease when cognitive decline starts to be evident while biomarker levels do not reach the cutoff criteria for diagnosing dementia [3], and (3) a dementia stage in which the pathology and symptomatology is fully developed [4]. The duration of each phase is not constant but rather depends on the age of onset, gender, and genetic risk factors [5].

The development of these stages is related to the spread of pathological changes across different brain regions. AD pathological hallmarks, comprising accumulation of amyloid-β peptide (Aβ) aggregates forming extracellular plaques, and hyperphosphorylated species of tau microtubule-associated protein, forming intraneuronal neurofibrillary tangles (NFTs) are known to follow an anatomical-temporal pattern starting in the temporal lobe and spreading to neocortical areas at later stages [6,7]. Although amyloid plaques are irregularly distributed in the brain, and their accumulation does not correlate with cognitive impairment [8], NFTs tau pathology progress pattern tends to be mostly maintained between patients [6]. Due to the stability of this pattern, NFTs pathology is the base of the most used pathological classification of the disease, established by Braak and Braak three decades ago [6]. The first two stages (I-II) could be associated with a preclinical phase of the disease, where tau pathology is mainly focused on the entorhinal cortex with subtle hippocampus affection [7,9] accompanied by synaptic function alteration. In stages III-IV the pathology affects the subcortical limbic region and clinically might correspond to the prodromal phase. Finally, during the last stages (V-VI), the pathology spreads to most neocortical areas [6,7] with observable extensive neuronal death, corresponding with AD dementia [6,9]. Increasing evidence in AD experimental models suggest that this progression pattern arises from trans-synaptic propagation of misfolded tau between established neural circuits [10].

Despite the huge amount of work done in the field during the last decades, and the significant advances achieved, our knowledge of the mechanisms underlying this multifactorial complex disease is still limited. This fact added to the lack of accessible and reliable methods for detecting preclinical phases has probably led to the failure of potential therapies so far [11]. In this regard, both preclinical and prodromal stages represent a potential therapeutic window where novel pharmacological and non-pharmacological therapies are more likely to delay the progression of the disease and improve the lives of patients. Therefore, it is urgent to improve the tools currently available to achieve a reliable and earlier detection of AD, ideally during initial phases characterized by alteration of synaptic function [12].

In this article, we will review the literature supporting the role of miRNAs in synaptic dysfunction, with special focus on their potential as early biomarkers for AD, both as diagnostic and prognostic tools.

## 2. Synaptic Function and Synaptic Alteration in AD

Changes in synapses’ structure and function involve gene regulatory networks controlling spine development, maturation, and maintenance. Interestingly, mutations in genes encoding synaptic proteins, and mutations in genes related to Aβ metabolism/clearance have been related to AD risk [13]. An increasing amount of evidence indicates that cognitive decline observed in the early stages of neurodegenerative diseases such as AD is a consequence of synaptic alterations that occurs before neurodegeneration takes place [14,15]. Since aberrant changes in dendritic spine morphology and density linked to altered number and function of neurotransmitter receptors contribute to synaptic failure in AD [15], better knowledge of both physiology and pathology of synapses is necessary to understand the mechanisms underlying AD pathology.

Synapses are dynamic structures whose correct function requires highly specialized molecular machinery at both pre- and post-synaptic terminals. Whereas pre-synaptic terminals are structurally similar in inhibitory and excitatory synapses, post-synaptic compartments differ in their organization [16]. Inhibitory synapses are typically located at the dendritic shaft or even at the neuronal soma where gephyrin anchors GABA receptors in the membrane. On the contrary, excitatory synapses containing glutamate receptors mostly rely on the dendritic spine structure, specialized protrusions of diverse sizes and shapes that allow a greater concentration of synapses in a compact area of the post-synaptic terminal [17]. Formed by a complex organization of scaffold proteins (including homer, shank, and PSD-95 protein families), the post-synaptic density (PSD) supports the structure of the post-synapse, anchoring ionotropic glutamate *N*-methyl-d-aspartate receptors (NMDAR) and synaptic α-amino-3-hydroxy-5-methylisoxazole-4-propionic acid receptors (AMPAR) as well as metabotropic glutamate receptors (mGluRs) in the cell surface together with a large number of signaling molecules and actin filaments [18].

Synapses are able to modify their structure and function upon neural activity, adapting the strength or efficacy of synaptic transmission to different contexts in a process known as synaptic plasticity [19]. Between several synaptic plasticity mechanisms that can occur in pre- and post-synaptic compartments, the most widely studied and understood are long-term potentiation (LTP) and long-term depression (LTD) processes in which both NMDAR and AMPAR could be involved. Initial Ca^2+^ influx through post-synaptic NMDAR regulates the recruitment or removal of synaptic *AMPAR*, reinforcing or weakening synaptic transmission, respectively, processes thought to underlie learning and memory functions [20,21].

The PSD-95 family is the most abundant post-synaptic scaffolding protein within the PSD containing three protein-protein interaction motifs (PDZ domains) that facilitate signal coupling by bringing together cytoplasmic signaling molecules such as kinases and phosphatases close to their substrate, controlling receptor assembling at synapses [18,22]. Different members of the PSD-95 family mediate AMPAR targeting to mature synapses supporting synaptic transmission and are also able to interact with transmembrane AMPAR regulatory proteins (TARP) stabilizing new synaptic AMPAR at the synapse [23]. Moreover, PSD-95 connects pre- and post-synaptic elements through the interaction with cell-adhesion molecules (CAMs) such as, neuroligins, neurexins, ephrins, or cadherins [24,25]. CAMs, at the same time, can be linked to F-actin cytoskeleton, giving a stable but flexible structure to the dendritic spines that underlies synaptic communication.

As expected, synaptic dysfunction observed in early AD patients is associated with extensive loss of synaptic markers [26,27] and a relationship between dysregulation of synaptic proteins and early cognitive dysfunction has been remarked [27,28]. Cognitive function evaluated by tests such as the Mini-Mental State Exam (MMSE) or the delayed word list recall tests that evaluate hippocampus-dependent tasks exhibit a positive correlation with the number of synapses in brain related areas as the hippocampus and frontal cortex of early AD patients [14,29].

Interestingly, synaptic density constitutes a better correlation with clinical symptoms than classical AD histopathological markers such as amyloid plaques and NFT [14,26,29,30]. In this regard, solid evidence indicates that oligomeric forms of Aβ (oAβ), instead of amyloid plaques, contribute to synaptic alterations and correlate with synaptic loss [12,31,32,33]. In the same line, it has been described that Aβ aggregation is enriched at synapses even before the formation of amyloid plaques or tau NFT [34].

Accordingly, oAβ are thought to initiate the pathological events described as the amyloid cascade hypothesis [35], according to which changes in Aβ metabolism result in its oligomerization that initially triggers disruption and loss of synaptic connections follow by inflammatory response involving microglial and astrocytic activation, alteration of calcium homeostasis and oxidative stress resulting in neurodegeneration. Indeed, oAβ were shown to alter pre-synaptic functions such as axonal transport, synaptic vesicles trafficking and recycling, and neurotransmitter release [36]. Moreover, evidence indicate that oAβ also affect post-synaptic function and are especially toxic to glutamatergic synapses. Dysregulation of glutamatergic transmission is the main described mechanism by which oAβ could alter spines shape and number [33,37,38,39,40] leading to synaptic failure [37]. For instance, the cascade triggered by oAβ affect the regulation of downstream kinases and phosphatases that increase NMDAR and AMPAR internalization [28,37,40], creating an imbalance between LTP and LTD. While these structural and functional alterations have been shown to contribute to cognitive dysfunction present in AD patients [41], several studies have also linked AMPAR decrease in the cell surface with synaptic alterations in AD experimental models, including primary neuronal cultures and transgenic mice models [28,42] in which, interestingly, a correlation also exists with learning and memory deficits [27,28]. Moreover, loss of synapses in the CA1 of transgenic mice models of AD are consistent with the synapse loss described in AD [15].

Furthermore, oAβ would facilitate tau phosphorylation, which in turn decreases its affinity for microtubules and facilitates its aggregation and NFTs formation contributing to signaling deficiency and neurodegeneration [43]. On the other hand, besides the well-known role of tau in microtubule assembly, studies in transgenic mice highlighted a dendritic role for tau during synaptic pathology [44,45,46]. During pathological processes, tau is able to bind to scaffolding proteins and glutamate receptors at the PSD affecting synaptic function by altering LTP-LTD balance [32]. In addition, tau has been shown to be necessary for Src-family tyrosine kinase Fyn recruitment to post-synaptic NMDAR complexes in a mechanism that mediates Aβ excitotoxicity [46]. Thus, both Aβ and tau constitute key pathogenic players in early neurodegenerative processes linked to AD [32], and they are thought to interact locally at synapses leading to synaptic failure and cognitive impairment [30,47,48].

## 3. miRNAs as Mediators of Synaptic Dysfunction in AD

Increasing evidence indicates that the alteration of protein functionality in the synapse could be involved in early synaptic alterations in AD [26,27]. Given the regulatory role of miRNAs, those miRNAs targeting synaptic-related proteins may be an important mechanism underlying the synaptic dysfunction present in early stages of the disease [49]. Thus, alterations in the levels of specific miRNAs could be important in the development of synaptic pathology that leads to neurodegeneration in AD by modifying the synaptic structure and function that underlie synaptic plasticity. In addition, miRNAs are capable of regulating the mechanisms of toxicity mediated by central factors of the pathology such as tau and Aβ. Moreover; miRNAs can be enveloped in membranous vesicles that can be released to the peripheral circulation in the form of extracellular vesicles such as exosomes, favoring miRNAs conservation [50]. In this context, it is reasonable to think that the possibility of detecting alteration in the levels of specific miRNAs during pathology constitutes a valuable tool for AD detection. Indeed, these facts have opened a very prolific field with an increasing number of candidates to be used as biomarkers for AD, among which, synaptic-related miRNAs seem the most promising candidates as biomarkers for the detection of early stages of AD since synaptic dysfunction precedes neurodegeneration and the clinical symptoms.

### 3.1. Synaptic Role of miRNAs

miRNAs are the most studied small noncoding RNAs (ncRNAs), a class of functional RNA molecules of approximately 22 nucleotides (nt) of length, lacking protein-coding properties. Over 2600 human mature miRNAs have been annotated so far (miRBase.org) and conserved miRNA-binding sites have been detected in more than 60 percent of protein-coding genes, suggesting a wide presence of miRNA-mediated gene expression regulation [51].

miRNAs post-transcriptionally regulate gene expression by associating with Argonaute (Ago) proteins to form the RNA-induced silencing complex (RISC). Binding by partial sequence complementarity usually to the 3-untranslated region (3′-UTR) of target messenger RNAs (mRNAs), a single miRNA could repress mRNA translation of hundreds of different targets [51,52] constituting a fine regulatory mechanism of protein expression. Although a repression of translation is the main mechanism of miRNAs regulation, other mechanisms including an increase in translation, have also been reported [53].

Many studies have shown the presence of specific miRNAs (including miR-9, miR-15b, miR-16, miR-135a/b miR-204, and miR-221) in axons and have related them to axon growth and branching [54,55,56]. Other studies have found highly expressed miRNAs (such as miR-9, miR-26a, miR-125b, miR-128, miR-132, miR-134, miR-138, miR-181a, or miR-218) within neuronal dendrites in diverse brain areas, including the hippocampus [55,57,58,59]. Importantly, not only mature miRNAs, but also precursor-miRNAs (pre-miRNA) and the RNAse III Dicer needed for miRNAs maturation, have been found within dendrites [58,60].

The enrichment of specific miRNAs in axons and dendrites indicates a potential role for miRNAs locally regulating protein levels, thus synaptic structure and function. In this regard, an elegant study by Erin M. Schuman and colleagues has demonstrated that synaptic activity can increase the processing of pre-miRNAs locally in dendrites. In particular, local maturation of miR-181a and subsequent decrease in its target CaMKIIα has been described [61]. Local regulation of Ca^2+^ signaling cascades related to learning and memory processes following neuronal activity through this key kinase, would constitute a fast local mechanism of synaptic function regulation by miRNAs [61].

Of particular interest, several transcription factors and co-activators such as the cyclic adenosine monophosphate (cAMP) response element-binding protein (CREB), CREB- regulated transcription coactivator-1 (CRTC1), and nuclear factor-kappa-light-chain-enhancer of activated B cells (NF-κB), have been related to AD. Interestingly, some of these transcriptional regulators are synaptonuclear factors implicated in regulating transcriptional programs related to synaptic function, often in an activity-dependent manner and through integration of signals mediated by CREB [62]. This transcription factor plays an essential role in activating transcriptional programs underlying synaptic plasticity [19]. Thereby, miRNAs binding to transcription factors would be able to regulate synaptic function. In this regard, Gerhard M. Schratt and colleagues have reported that CREB and myocyte enhancing factor 2 (MEF2) (a transcription factor that negatively regulates excitatory synapses’ number) regulate the expression of miR-212/132 family where miR-132 and miR-134 are included. Conversely, these miRNAs and other members of the family regulate CREB, SIRT, and the methyl CpG-binding protein 2 (MeCP2) expressions that can in turn regulate BDNF levels, triggering the induction of miR-212/132 expression. Indeed, miR-132 can bind directly to BDFN and regulate its expression. This complex regulatory loop constitutes a mechanism by which miRNAs can control their own levels in response to neural activity changes [63].

The specific case of miR-134, a brain specific miRNA and activity regulated member of this family, has been largely studied by Schratt et al. [64,65]. They have observed that miR-134 can be transported to dendrites as a mature miRNA, but it also exists as pre-miRNA in the synapto-dendritic compartment. Locally, miR-134 can mature when needed and can negatively regulate dendritic spines size targeting LIM domain kinase 1 (Limk1), involved in spine maturation. Interestingly, following synaptic activity, the repression that miR-134 exert over Limk1 translation is released, and dendritogenesis is promoted [64,65]. On the other hand, miR-134 can also facilitate homeostatic synaptic depression in response to chronic activity, targeting the local translational repressor Pumilio-2 [66]. Recently, miR-134 localization in dendrites has been supported using atomic force microscopy showing a negative correlation between the amount of miRNA and the maturity and function of synapses [49]. Other members of the family, miR-132 and miR-138, were also linked to synaptic formation and function [63].

Interestingly, miR-29a/b are known to bind to Arpc3 subunit of the ARP2/3 complex, involved in regulation of actin filament branching and dendritic spine morphogenesis [67], whereas miR-191 is known to target tropomodulin-2, a neuron-specific regulator of actin dynamics that decrease in response to increased miR-191 expression following NMDAR activity during LTD [68]. Similarly, miR-9 has been reported to target transcriptional repressor REST, thereby, acting as a mediator in mRNA translation activation, necessary for functions such as dendritic growth [69].

Since glutamatergic transmission plays a key role in synaptic function and plasticity processes, the study of miRNAs targeting excitatory synapses and specially glutamate receptors has caught special attention. For instance, the brain-enriched miR-9-3p above mentioned, targets SAP97 (a member of PSD-95-like membrane associated guanylate kinases-PSD-MAGUKs-) that binds to mRNA coding for the GluA1 AMPAR subunit, thus, modulating AMPAR trafficking [70]. Moreover, miR-92, miR-137, and miR-501 are able to selectively regulate GluA1 trafficking, so that their overexpression in vitro reduces AMPAR insertion in cell surface during homeostatic scaling [71,72,73]. In addition, other studies have indicated the direct regulation of GluA2 subunit by miR-124 [74], miR-218 [75] and miR-186 [76] in an activity-dependent manner. Of interest, Olde Loohuis and colleagues have reported that in addition to the regulation that miR-137 exerts on AMPAR-mediated synaptic transmission and the turnover between silent and active synapse, an activity dependent mechanism is in turn regulating miR-137 levels. Upregulation of miR-137 levels was observed following metabotropic glutamate receptor 5 (mGluR5) activation, leading to a negative feedback mechanism controlling mGluR5-dependent synaptic plasticity [73].

miRNAs regulating NMDAR expression have also been reported, for instance, miR-139-5p [77] and miR-125b [78] have been involved in synaptic plasticity regulation by binding to mRNA coding for NR2A subunit while miR-539 and miR-34a regulate NR2B subunit of NMDAR [79,80]. Moreover, indirect regulation of NMDAR through miR-128 binding to STIM2 (stromal interaction molecule 2) has also been suggested as a miRNA-mediated mechanism of synaptic plasticity modulation [81].

Regarding receptors regulation, the modulation of proteins related to receptor trafficking and function is also of great interest. For instance, miR-181c is predicted to target neuronal pentraxin 1 (NPTX1) and neuronal pentraxin receptor (NPTXR) while miR-210 has been shown to regulate the levels of both proteins [82] implicated in the modulation of glutamatergic transmission through modulation of AMPAR recruitment and clustering [83]. Interestingly, whereas miR-210 at the post-synaptic terminals is involved in the regulation of receptor trafficking and synaptic plasticity, at the pre-synaptic terminal, it has been shown to target SNAP25, participating in neurotransmitter release [84]. Regarding pre-synaptic function, miR-137 over-expression has been reported to alter synaptic vesicle docking accompanied by a decrease in active zone size and in synaptic vesicles number in hippocampal cultures, human-induced neurons, and in vivo experiments performed in mice [85]. Moreover, miR-485 has been associated with altered functional synapses number, spine maturation, PSD-95 clustering, and surface GluA2 expression by targeting the synaptic vesicle glycoprotein 2A (SV2A) involved in neurotransmitters release [86].

Given the central role of glutamatergic transmission in synaptic plasticity processes, the study of miRNAs targeting excitatory synapses, and especially glutamate receptors, has particularly grown during the last years. However, Katharine R. Smith and colleagues’ recent work has put the focus on GABAA receptor regulation by miR-376. In this study, a mechanism by which miR-376 locally inhibit *GABRA1* and *GABRG*2 translation in dendrites has been reported [87]. In this context, dendrite local *de novo* synthesis of synaptic GABAAR has been described, constituting a mechanism of long-term surface GABAAR clustering conservation during iLTP expression. Additionally, they described another level of regulation of this receptor, by which miR-376 is repressed through a calcineurin-NFAT-HDAC signaling pathway following NMDA-induced inhibitory long-term potentiation (iLTP), relieving miRNA-inhibition on GABAAR subunits translation.

**Figure 2:**
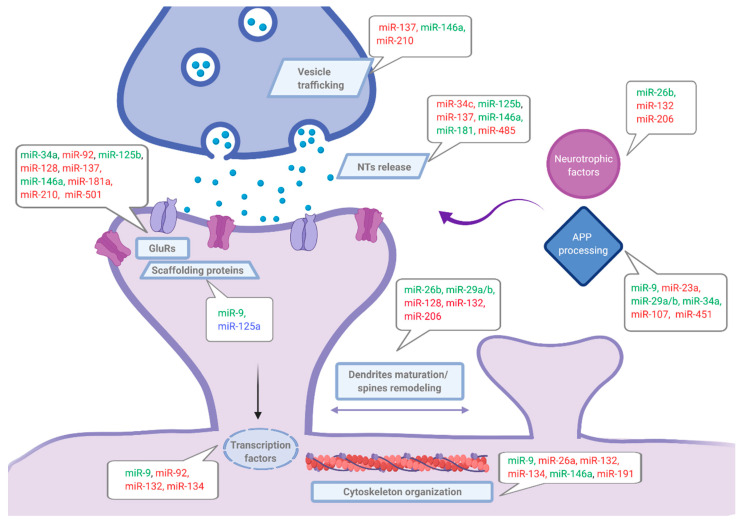
miRNAs regulating synaptic-related targets organized in key functional groups. Main altered miRNAs included in Table 1 and Table 2 and reported in blood (red), cerebrospinal fluid (CSF) (blue), or in both biofluids (green), are indicated. NTs: Neurotransmitters, GluRs: Glutamate receptors.

Data reviewed in this section (summarized in Table 1 and Figure 2) strongly support an essential role of miRNAs in synaptic plasticity modulation and consequently suggested their implication also in synaptic dysfunction related to neurodegenerative diseases. Certainly, understanding how specific miRNAs regulate synaptic function at different levels is essential to complete the full landscape of mechanisms underlying AD pathology, especially regarding synaptic failure present in the early stages of the disease.

### 3.2. miRNAs and Synaptic Dysfunction in AD

A large number of miRNAs have been shown to be dysregulated during AD progression [57,111] and in AD experimental models [89] expanding the role of miRNAs from physiology to pathology. Considering the central role that synaptic dysfunction plays in AD onset [30,47] the study of miRNAs particularly regulating synaptic function/dysfunction is of special interest and highlighting them constitutes the aim of this review.

Several of the miRNAs mentioned above, known to regulate synaptic proteins, have been suggested as important mediators of AD pathological processes (Table 1). For instance, miR-34a has been proposed as a potential contributor to AD pathology since its increased expression has been observed in APP/PS1 transgenic mice prior to Aβ increase, deposition into plaques, and cognitive deficits (appearing later in this model). In contrast, synaptic plasticity was shown to be enhanced in miR-34a-KO/APP/PS1 mice [109]. Besides multiple synaptic targets already described for this miRNA, including AMPAR and NMDAR, overexpression of miR-34a has been associated with a consistent reduction of sirtuin1 (SIRT1) [80]. Interestingly, other miRNAs, including miR-9 and miR-181c, have also been proposed to target SIRT1 [93] constituting interesting candidates of synaptic regulation in the context of AD pathology. Whereas miR-34a overexpression has also been reported to block tau synthesis by binding directly to the 3′-UTR of human tau mRNA [112], another member of the same family, miR-34c, has been reported to be upregulated in a transgenic AD model and in hippocampal neurons exposed to Aβ. Aβ toxicity was able to alter synaptic vesicle exocytosis through a miR-34c-mediated reduction of VAMP2, a protein component of the SNARE complex. These changes were associated with synaptic failure and learning and memory deficits, recovered after miR-34c increase blockade [105].

miR-134-5p, a well-studied brain-specific miRNA in synaptic function, has been reported to be upregulated after hippocampal Aβ treatment in rats. Interestingly, these results were linked to miR-134-mediated post-transcriptional regulation of CREB and BDNF since knockdown of miR-134-5p abolished CREB and BDNF miR-134-mediated decrease and rescues Aβ induced synaptic deficits [113]. In this way, miR-134-5p could be involved in a main molecular mechanism underlying synaptic plasticity alterations associated to AD.

In addition, miR-206 increased in both Tg2576 mice, and AD brain has also been shown to regulate memory function in AD mice models by targeting BDNF [98]. miR-206 is also able to regulate insulin like growth factor 1 (IGF1) [114]; likewise, miR-26b was associated with an increase in Aβ production through inhibition of IGF1 translation in vitro [97] regulate neprilysin (NEP), a type II transmembrane glycoprotein found on the pre-synaptic membrane and which downregulation has been linked to Aβ increased levels in mice AD model [99].

Related to AMPAR, different subunits have been validated as targets for several miRNAs in vitro and linked to cognitive deficits in experimental rodent models of AD. This redundant targeting of different glutamate receptor subunits suggests a complex miRNA-mediated post-transcriptional regulation of AMPAR trafficking during homeostatic scaling and subsequent role in synaptic plasticity alterations present in AD. For instance, the downregulation of miR-181a in 3xTg-AD mice rescued memory impairment through restoration of GluA2 levels [110]. By regulating GluA2 transcripts, miR-181a participate in Aβ-induced synaptic alterations while in the hippocampus it has been shown to regulate soluble tau levels, constituting a negative regulator of synaptic plasticity [110]. Furthermore, miR-30b, miR-186-5p, and miR-218 have also been reported to bind *Gria*2 3′-UTR and their overexpression have been associated with a decrease in GluA2 levels regulating synaptic function and cognitive decline in AD experimental models [76,115]. Other miRNA targeting GluA2, miR-124, has been reported to be strongly increased in the hippocampus of AD patients and also in AD mice model (Tg2576) accompanied by deficits in synaptic transmission, plasticity, and memory impairment [116]. Mechanisms underlying these synaptic deficits were further studied in vitro using hippocampal neurons, where oAβ treatment produce a miR-124-mediated decrease of PTPN1 (protein tyrosine phosphatase non-receptor type 1), impairing GluA2 membrane insertion [116]. Moreover, studies in P301S mice indicate that miR-124/PTPN1 alterations could also be modulating tau phosphorylation state through kinase/phosphatase activity imbalance [112].

Taken together, these studies showed that levels of miRNAs regulating glutamate receptors and synaptic-related proteins are altered in AD patients and mouse models, suggesting that they may play key pathological roles, besides informing about early synaptic deficits during the disease progression.

### 3.3. Role of miRNAs in Aβ/Tau Mediated Synaptic Dysfunction and Neuroinflammation

An increasing amount of studies in the area have already provided evidence of miRNAs dysregulated in AD, which are implicated in regulating key genes involved in the disease onset, such as PSEN1, APP, or BACE1 [111,117].

Studies using experimental models of AD have allowed the identification of miRNAs directly linked to tau neuropathology in AD. Regulating tau phosphorylation, specific miRNAs can modulate tau affinity for microtubule, maintenance of microtubule network, and tau aggregation/deposition in NFTs [43,118,119]. For instance, miR-125b and miR-138, both frequently upregulated in AD, have been shown to increase tau hyperphosphorylation and aggregation in neuronal cultures [120] and to impair associative learning in fear conditioning test in mice model of AD [121]. Moreover, miR-132, consistently decreased in AD studies [57,122], has also been associated with tau phosphorylation state regulation [111]. Increased tau hyperphosphorylation and aggregation was observed in 3xTg-AD mice expressing lower levels of miR-132 accompanied by long-term memory deficits [123].

Regarding Aβ metabolism, several miRNAs including miR-9, miR-29, miR-107, miR-124, miR-135b, miR-188, and miR-338 have been suggested to regulate β-site APP cleaving enzyme 1 (BACE1) levels [88,89,90,91,92], a central enzyme in Aβ generation. One example is miR-29a/b/c cluster, known to be decreased in AD brain [88], which correlates with increased BACE levels. Interestingly, miR-29a/b overexpression in AD SAMP8 mice model has been related to BACE1 and Aβ decrease, accompanied by learning and memory recovery [124]. While miR-188-3p overexpression in transgenic AD mice reduces Aβ levels through BACE1 translation inhibition [125], an association with dendritic spines alteration and synaptic transmission deficiency by regulation of Neuropilin-2 (Nrp-2) was also reported [126]. This way, miR-188-3p would be able to modulate the expression of glutamate receptors [125].

Furthermore, miRNAs targeting directly APP have also been described supporting the role of miRNAs in AD pathogenesis. Specifically, miR-101 levels downregulation observed in AD brain [88] is consistent with in vitro studies where inhibition of miR-101 increase APP levels [127]. Moreover, miR-16 and miR-147 have also been described to target APP in experimental models of AD pathology [128,129,130]. Interestingly, miR-16 expression has also been related to tau phosphorylation in primary cortical neurons [131].

Moreover, several miRNAs have been suggested to regulate metalloproteinase ADAM10 levels, including miR-23a, miR-34a, miR-107, and miR-451 [80,132,133]. Since APP is one of the well-known substrates of ADAM10, these miRNAs would be involved in Aβ metabolism. Additionally, other substrates with potential synaptic effect including cell adhesion molecules such as Neuroligin-1, N-Cadherin or NrCAM, have also been established for ADAM10 [134] relating these miRNAs to synaptic deficits linked to AD.

Otherwise, it is known that reactive astrocytes and inflammatory microglia are part of the pathological AD environment, although outside the scope of this review, it is interesting to point out that both cell populations produce and secrete extracellular vesicles (EVs) that are able to fuse with target cells to which they transfer their content (containing protein, lipids, and RNA species among which miRNAs are the most abundant type). This physiological communication channel between different cell types is known to be altered in pathological conditions [135]. In this regard, EVs secreted from reactive microglia were shown to be enriched in specific miRNAs, including miR-146a-5p that can be transferred to neurons, thereby downregulating neuronal synaptic targets such as synaptotagmin1 (Syt1) and neuroligin1 (Nlg1), negatively affecting dendritic spine density and excitatory synapses reduction [106]. Of interest, miR-26a, enriched in astrocyte-derived EVs, can be transferred to hippocampal neurons modulating their dendritic complexity [50].

Therefore, besides regulating synaptic-related proteins and transcription factors implicated in synaptic plasticity, miRNAs targeting genes related to Aβ and tau metabolism and activity can also contribute to synaptic dysfunction during AD, which may be further modulated by glial cells during neuroinflammation.

## 4. miRNAs as Biomarkers of AD

### 4.1. Current Biomarkers for AD

The increasing incidence of AD project devastating numbers: 152 million people will be living with dementia by 2050 [136] while the annual cost of dementia will reach 1 trillion dollars. The massive impact of the disease on the health and economic systems, as well as on families and caregivers, raises the pressure to accelerate the search for effective treatments able to, at least, delay the disease progression, but also to validate diagnostic tools that enable earlier detection of the disease [136]. Since MCI patients converted to AD at an annual rate of 17.2 percent [137], an earlier diagnosis would allow a better scenario for potential therapies to succeed, avoiding the limitation of an environment where neuronal death is already widespread and allowing the inclusion in clinical studies of patients in early stages of the disease, increasing the possibility of achieving better therapeutic results.

Currently, the use of biomarkers that can identify AD during its asymptomatic phase [138,139,140] is limited to Aβ_1–42_/Tau levels in cerebrospinal fluid (CSF) and imaging techniques such as the positron emission tomography (PET) that allow the observation of Aβ accumulation in the brain or 18F-fluorodeoxyglucose-PET (FDG-PET) to detect the decrease in glucose metabolic rate. Even combination of imaging approaches at different stages of the diagnosis are being evaluated to improve the available diagnostic tools [141]. However, due to their invasiveness and/or cost, they are not able to be included in routine clinical screenings, needed for early detection. In addition, recent studies discourage the use of CSF Aβ levels as a tool for detecting MCI cases since the accuracy in these cases is lower than recommended [142].

A promising candidate for monitoring disease progression and treatment response in pre-clinical research is the Neurofilament Light Chain (NfL). Remarkably, work done on the Dominantly Inherited Alzheimer Network (DIAN) demonstrated that the increased NfL levels in serum and CSF predict disease progression and brain neurodegeneration at asymptomatic stages of familial AD [143]. Of importance, NfL would constitute a general marker of neurodegeneration regardless of the subjacent cause [144].

### 4.2. Synaptic-Related miRNAs as Early Biomarkers for AD

The potential value of other molecules present in biological fluids (including CSF, blood, urine, and tears) to differentiate between AD and control subjects are also under evaluation. In this regard, miRNAs, close modulators of the specific pathogenic processes underlying AD and present in all these circulating fluids, are presented as promising candidates. Easily measured by simple and affordable techniques, including quantitative real-time PCR (RT-qPCR), miRNAs detection would allow a cost-effective and non-invasive method that could certainly be included in screening studies and patient follow-up over time.

miRNAs dysregulated either in brain or circulating fluids during AD pathology have been described over the last years and are the subject of recent review articles [145,146]. Some miRNAs already commented in this review (including miR-26b, miR-34a/c, miR-125b, miR-146a, and miR-210) have been described to be altered in brain and blood from AD patients, although the direction of changes is not always consistent between both miRNA sources [145,146]. Moreover, miRNAs isolated from AD plasma and serum (including miR-107, miR342-3p, miR-15b-5p, miR-545-3p, and miR-191-5p) have been proposed as potential AD biomarkers [145]. In addition, miR-455-3p, related to Aβ toxicity modulation, has been proposed as potential AD biomarker since increased levels observed in serum are consistent with levels in AD brains, fibroblasts, lymphocytes, and even AD transgenic models. Furthermore, a panel of miRNAs involved in pathological processes underlying AD, such as neuroinflammation, has been proposed as a diagnostic tool to predict AD onset, since the ability of a single miRNA to detect prodromal AD has not been fully demonstrated [146].

While intensive research is being done on miRNA-based biomarkers for AD, the relationship between synaptic function regulation and AD biomarkers is not present in many studies in the field. Most relevant findings in synaptic-related miRNAs obtained from circulating biofluids of MCI and AD patients and their potential value as biomarkers are summarized in Table 2, where it is evident that most studies have been done in blood samples, including serum and plasma, indicating an interest to explore less invasive biomarkers.

For instance, miR-132 decrease in serum from MCI and AD patients [151,161] has been reproduced in plasma samples [160], however an increase has also been reported in MCI subjects by Sheinerman and colleagues [159], evidencing that reproducibility between studies can be difficult even when obtaining miRNAs from the same sample source. In the same study, they have also reported the increase of another member of the same family, miR-134, and remarkably, both miRNAs showed notable performance with an area under the curve (AUC) over 0.9 (close to the ideal value of 1, which represents the maximum accuracy of a classifier distinguishing between two groups [169] and sensitivity between 82% and 86% for detection of MCI cases. Interestingly, miR-132 has also been proposed as a member of a serum-based signature for MCI detection, together with miR-206, which is also downregulated in MCI serum [161]. With an outstanding AUC of 0.98, a sensitivity of 85%, and specificity of 98%, this synaptic-related miRNA signature seems certainly promising.

The use of miRNA-based signatures, which consider the alteration of more than one miRNA at the same time, can give higher accuracy, sensitivity, and specificity values that could be an advantage for potential diagnostic tools as exposed in Table 3.

In this regard, another signature based in four serum-miRNA levels, including synaptic-related miR-29a, miR-125b, and miR-23a has shown an encouraging performance for differentiating AD cases from healthy cognitive controls (HCC) [149]. The diagnostic value of miR-29a/b family has been tested both in serum and CSF [148,149,151,154,155] although results are not consistent between studies, the alteration of these miRNAs in biological fluids during AD pathology seems to be clear. On the other hand, miR-125b and miR-23a increase has been consistently observed in serum [149,158], displaying good capacity of differentiation between AD and control subjects specifically for miR-125b alone. The potential of miR-125b has also been tested in CSF [152,154], where it has been proposed as a specific tool since, in addition, it is able to distinguish AD from FTD patients [152]. An increase in related miR-125a levels has also been described in CSF from AD patients [151,155], constituting a potential biomarker as previously reported, with special value differentiating also AD from FTD patients [151].

Altered levels of miR-34a were observed both in plasma and CSF [94,154,156], and values near 0.8 were reported for the AUC for plasma-based miRNA performance as AD biomarker. Another member of miR-34 family, miR-34c, was reported to be increased in plasma samples from AD patients, and more interestingly, in mild AD patients [94]. Furthermore, both family members have been included in a signature combining miRNA and piRNAs (PIWI-interacting RNAs) that can distinguish AD cases with 83% accuracy. Although the great value of this signature is predicting the conversion of MCI to AD, this capacity is due to piRNAs levels [172].

Regarding longitudinal studies, to our knowledge, only a few articles have evaluated miRNA levels over time as a predictive tool of MCI progression to AD. Indeed, miR-206 (mentioned above for its potential to distinguish MCI patients) has also shown a good prognostic capacity to classify MCI progression to AD over five years [165]. In another study, miR-181a and miR-146a levels in blood from MCI patients have been documented as a useful tool to distinguish between MCI patients that progress to AD (pMCI) and those who remain stable in MCI state (sMCI) two years after the first measure [162]. The levels in serum of another member of the family, miR-181c, have also been reported to distinguish MCI and AD from control subjects, although the direction of level changes is inconsistent between studies [148,150,158]. Interestingly, an increase in miR-181c in plasma samples from MCI and AD patients has been reported to be specific compared with an FTD cohort [163]. Moreover, both miR-181a and miR-181c have been included in individual plasma-based miRNA signatures. The first one, composed of six miRNAs [170], also include miR-9, which levels have been reported to similarly be altered in whole-blood [173], serum [148], and exosome-enriched CSF [147] from AD patients compared to HCC. With a high AUC, sensitivity, and specificity, this signature can differentiate AD, PD, and FTD from HCC, constituting a useful tool for detecting general neurodegeneration. Interestingly, the signature including miR-181c, is composed of two other synaptic-related miRNAs: miR-92a-3p and miR-210-3p, and besides the potential for distinguishing MCI and AD patients from HCC, promising preliminary results were reported in the signature capacity for evaluating MCI progression to AD after a follow-up of the patients from 1 to 11 years [163]. The potential value of miR-92a as biomarker for AD has also been evaluated in serum, where its levels were shown to be decreased during AD pathology [151].

miR-26b consistent upregulation has been reported in serum and whole blood samples from AD patients [152,153,174], whereas downregulation of miR-26a [150] has been reported in a study that comprised one of the biggest cohorts of AD patients included in this review. While miR-26b levels in CSF samples exhibited specificity for AD detection with an AUC value of 0.82 and the capacity to distinguish AD from FTD patients [152]; differentiation between AD and FTD patients was not possible in serum despite a higher AUC (0.97) for AD cohort [151]. Interestingly, both members of the family were comprised in a blood-based miRNA signature with other 10 miRNAs [171] including miR-107, also proposed itself as plasma biomarker for MCI and AD detection. Remarkably, this signature was tested in other neurodegenerative diseases, such as MS and PD, exhibiting specificity for MCI and AD detection. Furthermore, while miR-26b was also included in a CSF-based signature together with miR-125b [152]; miR-26a was included in serum-based miRNA signature together with miR-181c and other four miRNAs. This last signature’s AUC, sensitivity, and specificity is the highest included in this review for detecting AD cases, with 0.99 accuracy, 93% sensitivity and 99% specificity [150].

miR-137 and miR-501, both known to target *GRIA1*, have been proposed as potential AD biomarkers after decreased levels identification in serum [148,168], while miR-128 [159] and miR-191 [164] have been proposed as plasma-based biomarkers for AD with an AUC performance over 0.95. miR-191 has been considered together with miR-15 for diagnostic value improvement, and slightly better results were achieved, especially for specificity [164].

Consistent downregulation of miR-146a levels was reported in serum, plasma, and CSF from AD patients [151,154]. Despite the small cohort size, downregulation of miR-451 was also observed in plasma EVs with high AUC value distinguishing AD cases from HCC and specific results compared to Dementia with Lewy bodies (DLB) plasma samples [166]. Increased levels of miR-485-3p were reported in AD serum compared to healthy controls, with an AUC value of 0.93 differentiating both groups [167].

### 4.3. Potential and Limitations of Synaptic-Related miRNAs as Early AD Biomarkers

As mentioned above, miRNAs could also be isolated from exosome-enriched or EVs fraction, though the best source for obtaining miRNAs-enriched samples is still unclear. Some studies propose that exosome-related miRNAs could represent a stable source of miRNAs over time and differentially expressed during pathological states [175,176], whereas other reports suggest that miRNAs expression within exosomes could be certainly low [177]. Even miRNAs included in vesicles and whole plasma cell-free miRNA profiles have been reported to be different [178]. The possibility of diverse miRNA sources, including CSF, plasma, and serum, is an advantage but also raises a problem when looking for reproducibility between studies. However, limitation goes beyond the above mentioned, since reported changes are often inconsistent within the same sample type, as observable in Table 2. The cohort size the parameters used for groups classification, subjects’ inclusion–exclusion criteria, miRNAs extraction and quantification methods, and statistical analysis performed, could make intricate the comparison between studies. Moreover, although the ROC curve analysis is not the only method available for testing diagnostic value, it is certainly the most used. However, the information given in some studies is limited to the significant or not significant differential expression between groups, which beyond the importance of this information; it does not determine the diagnostic value of a molecule itself.

The advantages and potential value of miRNAs as early biomarkers for AD highlight the urgent need for protocols standardization as an essential tool that would allow a faster progress in obtaining more reliable results in order to bring the advances to the clinics. Remarkably, molecular diagnostics companies, such as DiamiR, are already developing and commercializing miRNA-based technologies, constituting a reflection of the progress made in the area and the real possibilities of incorporating miRNAs as biomarkers for AD not only in screening and diagnosis but also as a valuable tool for improving definition of clinical trials participants.

## 5. miRNAs as Therapeutic Targets

In addition to the potential of miRNAs as biomarkers for AD, their capacity to regulate several targets related to the pathology suggest that they may be also considered as therapeutic targets. miRNAs can be modulated pharmacologically by administration of specific compounds (such as anti-inflammatory drugs) or by using antisense oligonucleotides or double-stranded synthetic oligonucleotide miRNAs that inhibit or mimic endogenous miRNAs function, respectively. Recent studies using in vitro approaches and AD animal models have shown that regulation of specific miRNAs may impact on neuroprotection, cognitive function, and neuronal regeneration (reviewed in [179]). Interestingly, some synaptic-related miRNAs discussed above, including miR-30a, miR-124, miR-128, and miR-146a, which regulate targets related to Aβ accumulation and synaptic dysfunction, have been suggested as potential therapeutic targets for AD [179]. In addition, miR-485, related to neurotransmitter release [86] and neuroinflammation [167], has also been proposed both as potential AD biomarker and therapeutic target [167]. Furthermore, multitargeted therapeutic strategies such as the combination of acetylcholinesterase (AChE) inhibitors and modulation of specific miRNAs are also under evaluation [180].

Two key limitations of approaching miRNAs as therapeutic targets are 1) their capacity to regulate several transcripts (up to hundreds of them) simultaneously, and 2) the difficulty to achieve efficient miRNA delivery. Regarding the latter, a recent study using engineered exosomes to deliver miR-29 in a rat model of induced Aβ pathology, showed a rescue of memory deficits [181].

## 6. Concluding Remarks

Substantial progress has been made in the study of miRNAs as biomarkers of AD, which is especially important in finding miRNAs capable of detecting asymptomatic stages of the disease. In this review, we highlighted the huge potential of synaptic-related miRNAs in this regard; nonetheless, much remains to be explored. For instance, the potential of miRNAs for predicting MCI conversion into AD dementia has only been explored in a few studies. Therefore, it is necessary to widely perform longitudinal studies in future research. Screening of pre-existing blood banks could be a valuable source of material for studies that aim to elucidate the alteration of specific miRNAs during the pathology progression. This kind of studies would allow the acquisition of an immense amount of data on the evolution of specific miRNAs levels over the years in healthy controls and MCI and AD patients, which would allow not only to confirm the significance of miRNAs in AD diagnosis but also to probe their potential as a valuable prognostic tool.

## Figures and Tables

**Table 1:** Key synaptic-related targets and reported miRNAs regulating them.

Key Targets	Relevant Function	Related-miRNAs	Ref. Related miRNAs
*BACE1*	TNF-α, ephrin-A2, and APP cleavage	miR-9, miR-107, miR-29a/b	[88,89,90,91,92]
*SIRT1*	Acetylation of substrates related to learning and memory	miR-9, miR-34a/c, miR-181c, miR-132	[93,94]
*ADAM10*	Non-amyloidogenic APP processing	miR-23a, miR-34a, miR-107, miR-451	[80,93]
*GSK3B*	Phosphorylation of key related targets including tau	miR-26a	[95]
*MEF2D*	Transcription factor involved in structural plasticity	miR-92	[96]
*CREB1*	Transcription factor involved in synaptic plasticity	miR-132, miR-134	[63]
*MECP2*	Transcription factor involved in synaptic plasticity	miR-132	[63]
*REST*	Gene silencing transcription factor involved in synaptogenesis, synaptic plasticity, and structural remodeling	miR-9	[69]
*IGF1*	Growth factor involved in synapse maturation	miR-26b, miR-206	[97]
*BDNF*	Neurotrophic factor involved in synaptic plasticity	miR-132, miR-206	[98]
*MME* (NEP)	Neurite outgrowth	miR-26b	[99]
*EFNA3*	(Ephrin-A3) Axon guidance	miR-210	[82]
*DCX*	Axon assembly and branching	miR-29a	[100]
*ARPP21*	Dendritic branching	miR-128	[101]
p250GAP	Actin reorganization in dendritic spines.	miR-132	[102]
*LIMK1*	Actin cytoskeleton organization	miR-134	[64,65]
*PUM2*	Actin cytoskeleton organization	miR-134	[66]
*TMOD2*	Actin filaments organization	miR-191	[68]
*DPYSL2*	(CRMP-2) Axon guidance	miR-181c	[103]
*SNAP25*	Vesicle trafficking	miR-210	[84]
*SV2A*	Neurotransmitter release	miR-485	[86]
*SYN2*	Neurotransmitter release	miR-125b, miR-181	[104]
*VAMP2*	Neurotransmitter release	miR-34c	[105]
*SYT1*	Vesicle trafficking/Neurotransmitter release	miR-137, miR-146a	[106]
*ARPC3*	Negatively regulates synaptic scaling	miR-29a/b	[67]
*MAP2*	Microtubules assembly	miR-26a	[50]
*MAP1B*	Microtubules stabilization	miR-9, miR-146a	[54,107]
*DLG4*	(PSD-95) Scaffold protein	miR-125a	[108]
*NLGN1*	AMPAR clustering, synaptic transmission	miR-146a	[106]
*CAMK2A*	Kinase involved in synaptic plasticity, neurotransmitter release and long-term potentiation	miR-181a	[61]
*SAP97*	Scaffold protein	miR-9	[70]
*STIM2*	Negative regulator of NMDA-evoked intracellular Ca^2+^	miR-128	[81]
*NPTX1*	AMPAR recruitment/clustering, synaptic transmission	miR-210	[82]
*GRIA1*	Synaptic transmission	miR-92, miR-137, miR-501, miR-34a	[71,72,73,109]
*GRIA2*	Synaptic transmission	miR-181a	[110]
*GRIN2A*	Synaptic transmission	miR-125b	[77,78]
*GRIN2B*	Synaptic transmission	miR-34a	[80,109]

Key synaptic-related targets and their functions are indicated. miRNAs targeting them that will be further discussed as potential MCI-AD biomarkers (Table 2) are included.

**Table 2:** Synaptic-related miRNAs suggested as mild cognitive impairment (MCI)-Alzheimer’s disease (AD) biomarkers.

	Source	Cohort	↑↓	AUC	Sen/Spec (%)	NDD Tested Specific for AD?	Other Biomarkers	Cognitive Test	Ref.
# miR9	CSF exosomes	HCC = 10 + 18					CSF Aβ		[147]
		AD = 10 + 18	↑	N/A	N/A		CSF tau		
	Serum	HCC = 7						MMSE	[148]
		MCI = 7							
		AD = 7	↓	N/A	N/A				
# miR-23a	Serum	HCC = 15 + 30				PD and VD/No		MMSE	[149]
		AD = 15 + 30	↑	0.71	57/83				
# miR-26a	Serum	HCC = 9 + 86						MMSE	[150]
		AD = 19 + 121	↓	0.75	85/57				
# miR-26b	Serum	HCC = 44				FTD/No	CSF Aβ	MMSE	[151]
		AD = 48	↑	0.97	89/89		CSF tau		
	CSF	HCC = 18				FTD/Yes	CSF Aβ	MMSE	[152]
		AD = 22		0.82	N/A		CSF tau		
	Blood	HCC = 22						MMSE	[153]
		AD = 48	↑	0.81	N/A				
# miR-29a	Serum	HCC = 15 + 30						MMSE	[149]
		AD = 15 + 30	↑	0.71	43/97				
	Serum	HCC = 7						MMSE	[148]
		MCI = 7							
		AD = 7	↓	N/A	N/A				
	CSF	HCC = 10					CSF Aβ	MMSE	[154]
		AD = 10	↑	N/A	N/A		CSF tau		
	CSF	HCC = 20						MMSE	[155]
		AD = 18	↑	N/A	N/A				
miR-29b	Serum	HCC = 44				FTD/Yes	CSF Aβ	MMSE	[151]
		AD = 48	↑	0.83	93/-		CSF tau		
	Serum	HCC = 7						MMSE	[148]
		MCI = 7							
		AD = 7	↓	N/A	N/A				
	CSF	HCC = 10					CSF Aβ	MMSE	[154]
		AD = 10	↑	N/A	N/A		CSF tau		
# miR-34a	Plasma	HCC = 21 + 15				PD/Yes	CSF Aβ	MMSEGDS	[156]
		MCI = 21 + 15					CSF tau		
		AD = 21 + 15	↓	0.79	80/71		ApoE 4		
	Plasma	HCC = 27						MMSE	[94]
		AD = 25	↑	0.81	84/74				
	CSF and plasma	HCC = 10					CSF Aβ	MMSE	[154]
		AD = 10	↓	N/A	N/A		CSF tau		
miR-34c	Plasma	HCC = 27						MMSE	[94]
		AD = 25	↑	0.99	92/96				
miR-92	Serum	HCC = 44				FTD/No	CSF Aβ	MMSE	[151]
		AD = 48	↓	0.8	N/A		CSF tau		
miR-107	Plasma	HCC = 120				PD/No		MMSE	[157]
		AD = 120	↓	0.74	77/59				
miR-125a	CSF	HCC = 44				FTD/Yes	CSF Aβ	MMSE	[151]
		AD = 48	↑	0–84	74/82		CSF tau		
	CSF	HCC = 20						MMSE	[155]
		AD = 18	↑	N/A	N/A				
# miR-125b	Serum	HCC = 155						MMSE	[158]
		AD = 105	↑	0.85	80/68				
	# Serum	HCC = 15 + 30						MMSE	[149]
		AD = 15 + 30	↑	0.71	63/76				
	CSF	HCC = 18				FTD/Yes	CSF Aβ	MMSE	[152]
		AD = 22		0.82	N/A		CSF tau		
	CSF	HCC = 10					CSF Aβ	MMSE	[154]
		AD = 10	↓	N/A	N/A		CSF tau		
miR-128	Plasma	HCC = 50						MMSE	[159]
		MCI = 50	↑	0.97	84/96				
# miR-132	Plasma	HCC = 50						MMSE	[159]
		MCI = 50	↑	0.97	88/98				
	Plasma	HCC = 31					CSF Aβ CSF tau	MMSE	[160]
		AD-MCI = 16							
		AD = 16	↓	0.77	N/A				
	Serum	HCC = 44					CSF Aβ	MMSE	[151]
		AD = 48	↓	0.79	N/A		CSF tau		
	Serum	HCC = 76							[161]
		MCI = 66	↓	0.91	70/100				
miR-134	Plasma	HCC = 50						MMSE	[159]
		MCI = 50	↑	0.92	86/82				
miR-137	Serum	HCC = 7 MCI = 7 AD = 7	↓	N/A	N/A			MMSE	[148]
miR146a	Serum	HCC = 44				FTD/No	CSF Aβ	MMSE	[151]
		AD = 48	↓	0.87	N/A		CSF tau		
	Blood	sMCI = 25					CSF Aβ CSF tau ApoE MRI	MMSE GDS	[162]
		pMCI = 19	↑	N/A	N/A				
	CSF and plasma	HCC = 10					CSF Aβ	MMSE	[154]
		AD = 10	↓	N/A	N/A		CSF tau		
miR-181a	Blood	sMCI = 24					CSF Aβ CSF tau ApoE MRI	MMSE GDS	[162]
		pMCI = 17	↑	N/A	N/A				
# miR-181c	Serum	HCC = 9 + 86						MMSE	[150]
		AD = 19 + 121	↓	0.78	72/73				
	Serum	HCC = 155						MMSE	[158]
		AD = 105	↑	0.74	75/64				
	Plasma	HCC = 14 + 24	↑			FTD/Yes		MMSE GDS	[163]
		MCI = 26		0.84	85/86				
		AD = 56		0.77	70/86				
	Serum	HCC = 7						MMSE	[148]
		MCI = 7							
		AD = 7	↓	N/A	N/A				
# miR-191	Plasma	HCC = 20 + 17							[164]
		MCI = 9							
		AD = 11 + 20		0.95	95/76				
# miR-206	Serum	HCC = 76	↓						[161]
		MCI = 66		0.91	70/100				
	Plasma	HCC = 31 MCI = 30 AD = 25	↑	N/A	N/A			MMSE	[165]
# miR-210	Plasma	HCC = 14 + 24	↑			FTD/Yes		MMSE GDS	[163]
		MCI = 26		0.74	77/71				
		AD = 56		0.80	81/71				
miR-451	Plasma Evs	HCC = 15				DLB/Yes	ApoE	MMSE	[166]
		AD = 10	↓	0.95					
miR-485-3p	Serum	HCC = 62						MMSE	[167]
		AD = 89	↑	0.93	84/97				
miR-501	Serum	HCC = 22					ApoE	MMSE	[168]
		AD = 36	↓	0.82	53/100				

miRNAs also included in a molecular signature in Table 3 are indicated (#). Source of miRNAs, Evs: Extracellular vesicles. Discovery cohorts and validation cohorts (D + V) are shown when reported. Control subject are referred in all cases as HCC: Healthy cognitive controls. MCI: Mild cognitive impairment. AD: Alzheimer’s disease. sMCI: Stable MCI. pMCI: Progressor MCI. Sense of change in miRNAs levels are shown as arrows for increased levels ↑ and decreased levels ↓. AUC: Area under the curve, sensitivity and specificity values are included when available. If AUC available, values over 0.7 were considered for inclusion. If more than one diseased cohort, several AUC values are included and are shown in the same line corresponding to the group being compared to HCC. Other neurodegenerative diseases (NDD) tested in the same study are reported: PD: Parkinson’s disease, VD: Vascular dementia, FTD: Frontotemporal dementia DLB: Dementia with Lewy bodies. Specificity of miRNA changes for detecting MCI and/or AD subjects is indicated with YES; when miRNA changes are also present in other NDD, No is indicated. Cognitive tests used for patients’ inclusion in each cohort are indicated as MMSE: Mini-Mental State Examination and GDS: Geriatric Depression Scale.

**Table 3:** miRNA-based signatures including at least one synaptic-related miRNAs proposed as MCI-AD biomarker.

	Source	Cohort	AUC	Sen/Spec (%)	NDD Tested/Specific for AD?	Other Biomarkers	Cognitive Test	Ref.
**miR-9**/miR-874 miR-329/**miR-181a** miR-99/let-7e	Plasma	HC = 50 AD = 50	0.96	88/96	PD-FTD/No	CSF Aβ CSF tau	MMSE	[170]
**miR-23a, miR-29a, miR-125b,** miR-22	Serum	HCC = 30 AD = 30	0.84	80/72			MMSE	[149]
**miR-26a-5p, miR-107, miR-26b-5p**, miR-112, miR-161, let-7d-3p, miR-5010-3p, miR-1285-5p, miR-151a-3p, miR-103a-3p, miR-532-5p, let-7f-5p.	Blood	HCC = 22 + 21 MCI = 18 AD = 94	0.84 0.93	81/88 95/95	PD-MS/Yes		MMSE	[171]
**miR-206, miR-132**	Serum	HCC = 76 MCI = 66	0.98	85/98				[161]
**miR-30a-5p, miR-34c,** miR-27a-3p, piR_019949, piR_020364	CSF exosomes	HCC = 38 + 44 MCI = 17 AD = 23 + 19	0.83			CSF Aβ CSF tau	MMSE	[172]
**miR26b, miR125b**	CSF	HCC = 18 AD = 22	0.80			CSF Aβ CSF tau	MMSE	[152]
**miR-191,** miR-15b	Plasma	HCC = 20 + 17 MCI = 9 AD = 11 + 20	0.96	95/82			MMSE	[164]
**miR-92a-3p, miR-181c-5p, miR-210-3p**	Plasma	HCC = 14 + 24 MCI = 26 AD = 56	0.90 0.85	85/86 93/71	FTD/Yes		MMSE GDS	[163]
**miR-26a-5p, miR-181c-3p**, miR-126-5p, miR-22-3p, miR-148b-5p, miR-106b3p, miR-6119-5p, miR-1246 and miR-660-5p	Serum	HCC = 9 + 86 AD = 19 + 121	0.99	93/99			MMSE CDR	[150]

Synaptic-related miRNAs within each signature are highlighted in bold letters. Discovery cohorts and validation cohorts (D + V) are shown when reported. Control subject are referred in all cases as HCC: Healthy cognitive controls. MCI: Mild cognitive impairment. AD: Alzheimer’s disease. AUC: Area under the curve, sensitivity and specificity values for the combination of miRNAs in the signature are included when available. If more than one diseased cohort, several AUC values are included and are shown in the same line corresponding to the group being compared to HCC. Other neurodegenerative diseases (NDD) tested in the same study are reported: PD: Parkinson’s disease, MS: Multiple sclerosis, FTD: Frontotemporal dementia. Specificity of miRNA changes for detecting MCI and/or AD subjects is indicated with YES; when miRNA changes are also present in other tested NDD, No is indicated. Cognitive tests used for patients’ inclusion in each cohort are indicated as MMSE: Mini-Mental State Examination and GDS: Geriatric Depression Scale. CDR: Clinical dementia rating.

## Data Availability

No new data were created or analyzed in this study. Data sharing is not applicable to this article.

## References

[1] Prince M., Comas-Herrera A., Knapp M., Guerchet M., Karagiannidou M. (2016). World Alzheimer Report 2016 Improving Healthcare for People Living with Dementia Coverage, Quality and Costs Now and In the Future.

[2] World Health Organization Dementia Fact Sheet. http://www.who.int/news-room/fact-sheets/detail/dementia.

[3] Dubois B., Hampel H., Feldman H.H., Scheltens P., Aisen P., Andrieu S., Bakardjian H., Benali H., Bertram L., Blennow K. (2016). Preclinical Alzheimer’s disease: Definition, natural history, and diagnostic criteria. Alzheimer’s Dement..

[4] Jack C.R., Bennett D.A., Blennow K., Carrillo M.C., Dunn B., Haeberlein S.B., Holtzman D.M., Jagust W., Jessen F., Karlawish J. (2018). NIA-AA Research Framework: Toward a biological definition of Alzheimer’s disease. Alzheimers Dement..

[5] Vermunt L., Sikkes S.A.M., van den Hout A., Handels R., Bos I., van der Flier W.M., Kern S., Ousset P.-J., Maruff P., Skoog I. (2019). Duration of preclinical, prodromal, and dementia stages of Alzheimer’s disease in relation to age, sex, and APOE genotype. Alzheimer’s Dement..

[6] Braak H., Braak E. (1991). Neuropathological stageing of Alzheimer-related changes. Acta Neuropathol..

[7] Hyman B.T., Van Hoesen G.W., Damasio A.R., Barnes C.L. (1984). Alzheimer’s disease: Cell-specific pathology isolates the hippocampal formation. Science.

[8] Querfurth H.W., LaFerla F.M. (2010). Alzheimer’s Disease. N. Engl. J. Med..

[9] Braak H., Alafuzoff I., Arzberger T., Kretzschmar H., Del Tredici K. (2006). Staging of Alzheimer disease-associated neurofibrillary pathology using paraffin sections and immunocytochemistry. Acta Neuropathol..

[10] Pooler A.M., Polydoro M., Wegmann S., Nicholls S.B., Spires-Jones T.L., Hyman B.T. (2013). Propagation of tau pathology in Alzheimer’s disease: Identification of novel therapeutic targets. Alzheimers Res. Ther..

[11] Cummings J.L., Morstorf T., Zhong K. (2014). Alzheimer’s disease drug-development pipeline: Few candidates, frequent failures. Alzheimer’s Res. Ther..

[12] Walsh D.M., Klyubin I., Fadeeva J.V., Cullen W.K., Anwyl R., Wolfe M.S., Rowan M.J., Selkoe D.J. (2002). Naturally secreted oligomers of amyloid β protein potently inhibit hippocampal long-term potentiation in vivo. Nature.

[13] Lambert J.C., Heath S., Even G., Campion D., Sleegers K., Hiltunen M., Combarros O., Zelenika D., Bullido M.J., Tavernier B. (2009). Genome-wide association study identifies variants at CLU and CR1 associated with Alzheimer’s disease. Nat. Genet..

[14] DeKosky S.T., Scheff S.W. (1990). Synapse loss in frontal cortex biopsies in Alzheimer’s disease: Correlation with cognitive severity. Ann. Neurol..

[15] Androuin A., Potier B., Nägerl U.V., Cattaert D., Danglot L., Thierry M., Youssef I., Triller A., Duyckaerts C., El Hachimi K.H. (2018). Evidence for altered dendritic spine compartmentalization in Alzheimer’s disease and functional effects in a mouse model. Acta Neuropathol..

[16] Sheng M., Hoogenraad C.C. (2007). The Postsynaptic Architecture of Excitatory Synapses: A More Quantitative View. Annu. Rev. Biochem..

[17] Harris K.M., Kater S.B. (1994). Dendritic spines: Cellular specializations imparting both stability and flexibility to synaptic function. Annu. Rev. Neurosci..

[18] O’brien R.J., Xu D., Petralia R.S., Steward O., Huganir R.L., Worley P. (1999). Synaptic Clustering of AMPA Receptors by the Extracellular Immediate-Early Gene Product Narp. Neuron.

[19] Kandel E.R. (2012). The molecular biology of memory: CAMP, PKA, CRE, CREB-1, CREB-2, and CPEB. Mol. Brain..

[20] Citri A., Malenka R.C. (2008). Synaptic plasticity: Multiple forms, functions, and mechanisms. Neuropsychopharmacology.

[21] Huganir R.L., Nicoll R.A. (2013). AMPARs and synaptic plasticity: The last 25 years. Neuron.

[22] Kim E., Sheng M. (2004). PDZ domain proteins of synapses. Nat. Rev. Neurosci..

[23] Chowdhury D., Hell J.W. (2018). Homeostatic synaptic scaling: Molecular regulators of synaptic AMPA-type glutamate receptors. F1000Research.

[24] Hruska M., Henderson N.T., Xia N.L., Le Marchand S.J., Dalva M.B. (2015). Anchoring and synaptic stability of PSD-95 is driven by ephrin-B3. Nat. Neurosci..

[25] Dean C., Dresbach T. (2006). Neuroligins and neurexins: Linking cell adhesion, synapse formation and cognitive function. Trends Neurosci..

[26] Masliah E., Mallory M., Alford M., DeTeresa R., Hansen L.A., McKeel D.W., Morris J.C. (2001). Altered expression of synaptic proteins occurs early during progression of Alzheimer’s disease. Neurology.

[27] Reddy P.H., Mani G., Park B.S., Jacques J., Murdoch G., Whetsell W., Kaye J., Manczak M. (2005). Differential loss of synaptic proteins in Alzheimer’s disease: Implications for synaptic dysfunction. J. Alzheimer’s Dis..

[28] Miñano-Molina A.J., España J., Martín E., Barneda-Zahonero B., Fadó R., Solé M., Trullás R., Saura C.A., Rodríguez-Alvarez J. (2011). Soluble oligomers of amyloid-β peptide disrupt membrane trafficking of α-amino-3-hydroxy-5-methylisoxazole-4-propionic acid receptor contributing to early synapse dysfunction. J. Biol. Chem..

[29] Scheff S.W., Price D.A., Schmitt F.A., DeKosky S.T., Mufson E.J., Rubin E.H., Morris J.C. (2007). Synaptic alterations in CA1 in mild Alzheimer disease and mild cognitive impairment. Neurology.

[30] Selkoe D.J. (2002). Alzheimer’s disease is a synaptic failure. Science.

[31] Pickett E.K., Koffie R.M., Wegmann S., Henstridge C.M., Herrmann A.G., Colom-Cadena M., Lleo A., Kay K.R., Vaught M., Soberman R. (2016). Non-Fibrillar Oligomeric Amyloid-within Synapses. J. Alzheimer’s Dis..

[32] Ittner A., Ittner L.M. (2018). Dendritic Tau in Alzheimer’s Disease. Neuron.

[33] Townsend M., Shankar G.M., Mehta T., Walsh D.M., Selkoe D.J. (2006). Effects of secreted oligomers of amyloid β-protein on hippocampal synaptic plasticity: A potent role for trimers. J. Physiol..

[34] Bilousova T., Miller C.A., Poon W.W., Vinters H.V., Corrada M., Kawas C., Hayden E.Y., Teplow D.B., Glabe C., Albay R. (2016). Synaptic amyloid-b oligomers precede p-tau and differentiate high pathology control cases. Am. J. Pathol..

[35] Hardy J.A., Higgins G.A. (1992). Alzheimer’s Disease: The Amyloid Cascade Hypothesis. Science.

[36] Ovsepian S.V., O’Leary V.B., Zaborszky L., Ntziachristos V., Dolly J.O. (2018). Synaptic vesicle cycle and amyloid β: Biting the hand that feeds. Alzheimer’s Dement..

[37] Hsieh H., Boehm J., Sato C., Iwatsubo T., Tomita T., Sisodia S., Malinow R. (2006). AMPAR Removal Underlies Aβ-Induced Synaptic Depression and Dendritic Spine Loss. Neuron.

[38] Parameshwaran K., Dhanasekaran M., Suppiramaniam V. (2008). Amyloid beta peptides and glutamatergic synaptic dysregulation. Exp. Neurol..

[39] Lacor P.N., Buniel M.C., Furlow P.W., Sanz Clemente A., Velasco P.T., Wood M., Viola K.L., Klein W.L. (2007). Ab Oligomer-Induced Aberrations in Synapse Composition, Shape, and Density Provide a Molecular Basis for Loss of Connectivity in Alzheimer’s Disease. J. Neurosci..

[40] Snyder E.M., Nong Y., Almeida C.G., Paul S., Moran T., Choi E.Y., Nairn A.C., Salter M.W., Lombroso P.J., Gouras G.K. (2005). Regulation of NMDA receptor trafficking by amyloid-β. Nat. Neurosci..

[41] Wang Z.-X., Tan L., Liu J., Yu J.-T. (2016). The Essential Role of Soluble Aβ Oligomers in Alzheimer’s Disease. Mol. Neurobiol..

[42] Baglietto-Vargas D., Prieto G.A., Limon A., Forner S., Rodriguez-Ortiz C.J., Ikemura K., Ager R.R., Medeiros R., Trujillo-Estrada L., Martini A.C. (2018). Impaired AMPA signaling and cytoskeletal alterations induce early synaptic dysfunction in a mouse model of Alzheimer’s disease. Aging Cell.

[43] Wang Y., Mandelkow E. (2016). Tau in physiology and pathology. Nat. Rev. Neurosci..

[44] Roberson E.D., Scearce-Levie K., Palop J.J., Yan F., Cheng I.H., Wu T., Gerstein H., Yu G.-Q., Mucke L. (2007). Reducing endogenous tau ameliorates amyloid beta-induced deficits in an Alzheimer’s disease mouse model. Science.

[45] Roberson E.D., Halabisky B., Yoo J.W., Yao J., Chin J., Yan F., Wu T., Hamto P., Devidze N., Yu G.-Q. (2011). Amyloid- /Fyn-Induced Synaptic, Network, and Cognitive Impairments Depend on Tau Levels in Multiple Mouse Models of Alzheimer’s Disease. J. Neurosci..

[46] Ittner L.M., Ke Y.D., Delerue F., Bi M., Gladbach A., van Eersel J., Wölfing H., Chieng B.C., Christie M.J., Napier I.A. (2010). Dendritic function of tau mediates amyloid-β toxicity in alzheimer’s disease mouse models. Cell.

[47] Walsh D.M., Selkoe D.J. (2004). Deciphering the molecular basis of memory failure in Alzheimer’s disease. Neuron.

[48] Miller E.C., Teravskis P.J., Dummer B.W., Zhao X., Huganir R.L., Liao D. (2014). Tau phosphorylation and tau mislocalization mediate soluble Ab oligomer-induced AMPA glutamate receptor signaling deficits. Eur. J. Neurosci..

[49] Park I., Kim H.J., Kim Y., Hwang H.S., Kasai H., Kim J.H., Park J.W. (2019). Nanoscale imaging reveals miRNA-mediated control of functional states of dendritic spines. Proc. Natl. Acad. Sci. USA.

[50] Luarte A., Henzi R., Fernández A., Gaete D., Cisternas P., Pizarro M., Batiz L.F., Villalobos I., Masalleras M., Vergara R. (2020). Astrocyte-Derived Small Extracellular Vesicles Regulate Dendritic Complexity through miR-26a-5p Activity. Cells.

[51] Daugaard I., Hansen T.B. (2017). Biogenesis and Function of Ago-Associated RNAs. Trends Genet..

[52] Filipowicz W., Bhattacharyya S.N., Sonenberg N. (2008). Mechanisms of post-transcriptional regulation by microRNAs: Are the answers in sight?. Nat. Rev. Genet..

[53] Vasudevan S., Steitz J.A. (2007). AU-Rich-Element-Mediated Upregulation of Translation by FXR1 and Argonaute 2. Cell.

[54] Dajas-Bailador F., Bonev B., Garcez P., Stanley P., Guillemot F., Papalopulu N. (2012). microRNA-9 regulates axon extension and branching by targeting Map1b in mouse cortical neurons. Nat. Neurosci..

[55] Olde Loohuis N.F.M., Kos A., Martens G.J.M., Van Bokhoven H., Nadif Kasri N., Aschrafi A. (2012). MicroRNA networks direct neuronal development and plasticity. Cell Mol. Life Sci..

[56] van Battum E.Y., Verhagen M.G., Vangoor V.R., Fujita Y., Derijck A.A.H.A., O’Duibhir E., Giuliani G., de Gunst T., Adolfs Y., Lelieveld D. (2018). An Image-Based miRNA Screen Identifies miRNA-135s As Regulators of CNS Axon Growth and Regeneration by Targeting Krüppel-like Factor 4. J. Neurosci..

[57] Cogswell J.P., Ward J., Taylor I.A., Waters M., Shi Y., Cannon B., Kelnar K., Kemppainen J., Brown D., Chen C. (2008). Identification of miRNA changes in Alzheimer’s disease brain and CSF yields putative biomarkers and insights into disease pathways. J. Alzheimer’s Dis..

[58] Lugli G., Torvik V.I., Larson J., Smalheiser N.R. (2008). Expression of microRNAs and their precursors in synaptic fractions of adult mouse forebrain. J. Neurochem..

[59] Kye M.-J., Liu T., Levy S.F., Xu N.L., Groves B.B., Bonneau R., Lao K., Kosik K.S. (2007). Somatodendritic microRNAs identified by laser capture and multiplex RT-PCR. RNA.

[60] Lugli G., Larson J., Martone M.E., Jones Y., Smalheiser N.R. (2003). Dicer and eIF2c are enriched at postsynaptic densities in adult mouse brain and are modified by neuronal activity in a calpain-dependent manner. J. Neurochem..

[61] Sambandan S., Akbalik G., Kochen L., Rinne J., Kahlstatt J., Glock C., Tushev G., Alvarez-Castelao B., Heckel A., Schuman E.M. (2017). Activity-dependent spatially localized miRNA maturation in neuronal dendrites. Science.

[62] Parra-Damas A., Saura C.A. (2019). Synapse-to-Nucleus Signaling in Neurodegenerative and Neuropsychiatric Disorders. Biol. Psychiatry.

[63] Bicker S., Lackinger M., Weiß K., Schratt G. (2014). MicroRNA-132, -134, and -138: A microRNA troika rules in neuronal dendrites. Cell Mol. Life Sci..

[64] Schratt G.M., Tuebing F., Nigh E.A., Kane C.G., Sabatini M.E., Kiebler M., Greenberg M.E. (2006). A brain-specific microRNA regulates dendritic spine development. Nature.

[65] Zampa F., Bicker S., Schratt G. (2018). Activity-Dependent Pre-miR-134 Dendritic Localization Is Required for Hippocampal Neuron Dendritogenesis. Front. Mol. Neurosci..

[66] Fiore R., Rajman M., Schwale C., Bicker S., Antoniou A., Bruehl C., Draguhn A., Schratt G. (2014). MiR-134-dependent regulation of Pumilio-2 is necessary for homeostatic synaptic depression. EMBO J..

[67] Lippi G., Steinert J.R., Marczylo E.L., D’Oro S., Fiore R., Forsythe I.D., Schratt G., Zoli M., Nicotera P., Young K.W. (2011). Targeting of the Arpc3 actin nucleation factor by miR-29a/b regulates dendritic spine morphology. J. Cell Biol..

[68] Hu Z., Yu D., Gu Q.H., Yang Y., Tu K., Zhu J., Li Z. (2014). MiR-191 and miR-135 are required for long-lasting spine remodelling associated with synaptic long-term depression. Nat. Commun..

[69] Giusti S.A., Vogl A.M., Brockmann M.M., Vercelli C.A., Rein M.L., Trümbach D., Wurst W., Cazalla D., Stein V., Deussing J.M. (2014). MicroRNA-9 controls dendritic development by targeting REST. Elife.

[70] Sim S.-E., Lim C.-S., Kim J.-I., Seo D., Chun H., Yu N.-K., Lee J., Kang S.J., Ko H.-G., Choi J.-H. (2016). The Brain-Enriched MicroRNA miR-9-3p Regulates Synaptic Plasticity and Memory. J. Neurosci..

[71] Letellier M., Elramah S., Mondin M., Soula A., Penn A., Choquet D., Landry M., Thoumine O., Favereaux A. (2014). miR-92a regulates expression of synaptic GluA1-containing AMPA receptors during homeostatic scaling. Nat. Neurosci..

[72] Hu Z., Zhao J., Hu T., Luo Y., Zhu J., Li Z. (2015). miR-501-3p mediates the activity-dependent regulation of the expression of AMPA receptor subunit GluA1. J. Cell Biol..

[73] Olde Loohuis N.F.M., Ba W., Stoerchel P.H., Kos A., Jager A., Schratt G., Martens G.J.M., van Bokhoven H., Nadif Kasri N., Aschrafi A. (2015). MicroRNA-137 Controls AMPA-Receptor-Mediated Transmission and mGluR-Dependent LTD. Cell Rep..

[74] Hou Q., Ruan H., Gilbert J., Wang G., Ma Q., Yao W.-D., Man H.-Y. (2015). MicroRNA miR124 is required for the expression of homeostatic synaptic plasticity. Nat. Commun..

[75] Rocchi A., Moretti D., Lignani G., Colombo E., Scholz-Starke J., Baldelli P., Tkatch T., Benfenati F. (2019). Neurite-Enriched MicroRNA-218 Stimulates Translation of the GluA2 Subunit and Increases Excitatory Synaptic Strength. Mol. Neurobiol..

[76] Silva M.M., Rodrigues B., Fernandes J., Santos S.D., Carreto L., Santos M.A.S., Pinheiro P., Carvalho A.L. (2019). MicroRNA-186-5p controls GluA2 surface expression and synaptic scaling in hippocampal neurons. Proc. Natl. Acad. Sci. USA.

[77] Alsharafi W.A., Xiao B., Li J. (2016). MicroRNA-139-5p negatively regulates NR2A-containing NMDA receptor in the rat pilocarpine model and patients with temporal lobe epilepsy. Epilepsia.

[78] Edbauer D., Neilson J.R., Foster K.A., Wang C.-F., Seeburg D.P., Batterton M.N., Tada T., Dolan B.M., Sharp P.A., Sheng M. (2010). Regulation of Synaptic Structure and Function by FMRP-Associated MicroRNAs miR-125b and miR-132. Neuron.

[79] Corbel C., Hernandez I., Wu B., Kosik K.S. (2015). Developmental attenuation of N-methyl-D-aspartate receptor subunit expression by microRNAs. Neural Dev..

[80] Sarkar S., Engler-Chiurazzi E.B., Cavendish J.Z., Povroznik J.M., Russell A.E., Quintana D.D., Mathers P.H., Simpkins J.W. (2019). Over-expression of miR-34a induces rapid cognitive impairment and Alzheimer’s disease-like pathology. Brain Res..

[81] Deng M., Zhang Q., Wu Z., Ma T., He A., Zhang T., Ke X., Yu Q., Han Y., Lu Y. (2020). Mossy cell synaptic dysfunction causes memory imprecision via miR-128 inhibition of STIM2 in Alzheimer’s disease mouse model. Aging Cell.

[82] Pulkkinen K., Malm T., Turunen M., Koistinaho J., Ylä-Herttuala S. (2008). Hypoxia induces microRNA miR-210 in vitro and in vivo. FEBS Lett..

[83] Pelkey K.A., Barksdale E., Craig M.T., Yuan X., Sukumaran M., Vargish G.A., Mitchell R.M., Wyeth M.S., Petralia R.S., Chittajallu R. (2015). Pentraxins coordinate excitatory synapse maturation and circuit integration of parvalbumin interneurons. Neuron.

[84] Ren Z., Yu J., Wu Z., Si W., Li X., Liu Y., Zhou J., Deng R., Chen D. (2018). MicroRNA-210-5p contributes to cognitive impairment in early vascular dementia rat model through targeting snap25. Front. Mol. Neurosci..

[85] Siegert S., Seo J., Kwon E.J., Rudenko A., Cho S., Wang W., Flood Z., Martorell A.J., Ericsson M., Mungenast A.E. (2015). The schizophrenia risk gene product miR-137 alters presynaptic plasticity. Nat. Neurosci..

[86] Cohen J.E., Lee P.R., Chen S., Li W., Fields R.D. (2011). MicroRNA regulation of homeostatic synaptic plasticity. Proc. Natl. Acad. Sci. USA.

[87] Rajgor D., Purkey A.M., Sanderson J.L., Welle T.M., Garcia J.D., Dell’Acqua M.L., Smith K.R. (2020). Local miRNA-Dependent Translational Control of GABAAR Synthesis during Inhibitory Long-Term Potentiation. Cell Rep..

[88] Hebert S.S., Horre K., Nicolai L., Papadopoulou A.S., Mandemakers W., Silahtaroglu A.N., Kauppinen S., Delacourte A., De Strooper B. (2008). Loss of microRNA cluster miR-29a/b-1 in sporadic Alzheimer’s disease correlates with increased BACE1/ -secretase expression. Proc. Natl Acad Sci. USA.

[89] Schonrock N., Ke Y.D., Humphreys D., Staufenbiel M., Ittner L.M., Preiss T., Rgen Gö Tz J., Götz J. (2010). Neuronal microrna deregulation in response to Alzheimer’s disease amyloid-β. PLoS ONE.

[90] Wang W.-X., Rajeev B.W., Stromberg A.J., Ren N., Tang G., Huang Q., Rigoutsos I., Nelson P.T. (2008). The Expression of MicroRNA miR-107 Decreases Early in Alzheimer’s Disease and May Accelerate Disease Progression through Regulation of -Site Amyloid Precursor Protein-Cleaving Enzyme 1. J. Neurosci..

[91] Zhang Y., Xing H., Guo S., Zheng Z., Wang H., Xu D. (2016). MicroRNA-135b has a neuroprotective role via targeting of β-site APP-cleaving enzyme 1. Exp. Ther. Med..

[92] Fang M.R., Wang J., Zhang X.B., Geng Y., Hu Z., Rudd J.A., Ling S., Chen W., Han S. (2012). The miR-124 regulates the expression of BACE1/beta-secretase correlated with cell death in Alzheimer’s disease. Toxicol. Lett..

[93] Schonrock N., Humphreys D.T., Preiss T., Götz J. (2012). Target gene repression mediated by miRNAs miR-181c and miR-9 both of which are down-regulated by amyloid-β. J. Mol. Neurosci..

[94] Bhatnagar S., Chertkow H., Schipper H.M., Yuan Z., Shetty V., Jenkins S., Jones T., Wang E. (2014). Increased microRNA-34c abundance in Alzheimer’s disease circulating blood plasma. Front. Mol. Neurosci..

[95] Lucci C., Mesquita-Ribeiro R., Rathbone A., Dajas-Bailador F. (2020). Spatiotemporal regulation of GSK3β levels by miRNA-26a controls axon development in cortical neurons. Development.

[96] Vetere G., Barbato C., Pezzola S., Yuan Z., Shetty V., Jenkins S., Jones T., Wang E. (2014). Selective inhibition of miR-92 in hippocampal neurons alters contextual fear memory. Hippocampus.

[97] Liu H., Chu W., Gong L., Gao X., Wang W. (2016). MicroRNA-26b is upregulated in a double transgenic mouse model of Alzheimer’s disease and promotes the expression of amyloid-β by targeting insulin-like growth factor 1. Mol. Med. Rep..

[98] Lee S.-T., Chu K., Jung K.-H., Kim J.H., Huh J.-Y., Yoon H., Park D.-K., Lim J.-Y., Kim J.-M., Jeon D. (2012). miR-206 regulates brain-derived neurotrophic factor in Alzheimer disease model. Ann. Neurol..

[99] Chu T., Shu Y., Qu Y., Gao S., Zhang L. (2018). miR-26b inhibits total neurite outgrowth, promotes cells apoptosis and downregulates neprilysin in Alzheimer’s disease. Int. J. Clin. Exp. Pathol..

[100] Li H., Mao S., Wang H., Zen K., Zhang C., Li L. (2014). MicroRNA-29a modulates axon branching by targeting doublecortin in primary neurons. Protein Cell..

[101] Rehfeld F., Maticzka D., Grosser S., Knauff P., Eravci M., Vida I., Backofen R., Wulczyn F.G. (2018). The RNA-binding protein ARPP21 controls dendritic branching by functionally opposing the miRNA it hosts. Nat. Commun..

[102] Impey S., Davare M., Lasiek A., Fortin D., Ando H., Varlamova O., Obrietan K., Soderling T.R., Goodman R.H., Wayman G.A. (2010). An activity-induced microRNA controls dendritic spine formation by regulating Rac1-PAK signaling. Mol. Cell Neurosci..

[103] Zhou H., Zhang R., Lu K., Yu W., Xie B., Cui D., Jiang L., Zhang Q., Xu S. (2016). Deregulation of miRNA-181c potentially contributes to the pathogenesis of AD by targeting collapsin response mediator protein 2 in mice. J. Neurol Sci..

[104] Kos A., Olde Loohuis N., Meinhardt J., van Bokhoven H., Kaplan B.B., Martens G.J., Aschrafi A. (2016). MicroRNA-181 promotes synaptogenesis and attenuates axonal outgrowth in cortical neurons. Cell Mol. Life Sci..

[105] Hu S., Wang H., Chen K., Cheng P., Gao S., Liu J., Li X., Sun X. (2015). MicroRNA-34c Downregulation Ameliorates Amyloid-β-Induced Synaptic Failure and Memory Deficits by Targeting VAMP2. J. Alzheimer’s Dis..

[106] Prada I., Gabrielli M., Turola E., Iorio A., D’Arrigo G., Parolisi R., De Luca M., Pacifici M., Bastoni M., Lombardi M. (2018). Glia-to-neuron transfer of miRNAs via extracellular vesicles: A new mechanism underlying inflammation-induced synaptic alterations. Acta Neuropathol..

[107] Chen Y.L., Shen C.K.J. (2013). Modulation of mGluR-dependent MAP1B translation and AMPA receptor endocytosis by microRNA miR-146a-5p. J. Neurosci..

[108] Muddashetty R.S., Nalavadi V.C., Gross C., Yao X., Xing L., Laur O., Warren S.T., Bassell G.J. (2011). Reversible inhibition of PSD-95 mRNA translation by miR-125a, FMRP phosphorylation and mGluR signaling. Mol. Cell..

[109] Xu Y., Chen P., Wang X., Yao J., Zhuang S. (2018). miR-34a deficiency in APP/PS1 mice promotes cognitive function by increasing synaptic plasticity via AMPA and NMDA receptors. Neurosci. Lett..

[110] Rodriguez-Ortiz C.J., Prieto G.A., Martini A.C., Forner S., Trujillo-Estrada L., LaFerla F.M., Baglietto-Vargas D., Cotman C.W., Kitazawa M. (2020). miR-181a negatively modulates synaptic plasticity in hippocampal cultures and its inhibition rescues memory deficits in a mouse model of Alzheimer’s disease. Aging Cell.

[111] Lau P., Bossers K., Janky R., Salta E., Frigerio C.S., Barbash S., Rothman R., Sierksma A.S.R., Thathiah A., Greenberg D. (2013). Alteration of the microRNA network during the progression of Alzheimer’s disease. EMBO Mol. Med..

[112] Hou T.Y., Zhou Y., Zhu L.S., Wang X., Pang P., Wang D.Q., Liuyang Z.Y., Man H., Lu Y., Zhu L.Q. (2020). Correcting abnormalities in miR-124/PTPN1 signaling rescues tau pathology in Alzheimer’s disease. J. Neurochem..

[113] Baby N., Alagappan N., Dheen S.T., Sajikumar S. (2020). MicroRNA-134-5p inhibition rescues long-term plasticity and synaptic tagging/capture in an Aβ(1–42)-induced model of Alzheimer’s disease. Aging Cell.

[114] Liu T.J., Wang B., Li Q.X., Dong X.L., Han X.L., Zhang S.B. (2018). Effects of microRNA-206 and its target gene IGF-1 on sevoflurane-induced activation of hippocampal astrocytes in aged rats through the PI3K/AKT/CREB signaling pathway. J. Cell Physiol..

[115] Song Y., Hu M., Zhang J., Teng Z.Q., Chen C. (2019). A novel mechanism of synaptic and cognitive impairments mediated via microRNA-30b in Alzheimer’s disease. EBioMedicine.

[116] Wang X., Liu D., Huang H.Z., Wang Z.-H., Hou T.-Y., Yang X., Pang P., Wei N., Zhou Y.-F., Dupras M.-J. (2017). A Novel MicroRNA-124/PTPN1 Signal Pathway Mediates Synaptic and Memory Deficits in Alzheimer’s Disease. Biol. Psychiatry.

[117] Hébert S.S., Horré K., Nicolaï L., Bergmans B., Papadopoulou A.S., Delacourte A., De Strooper B. (2009). MicroRNA regulation of Alzheimer’s Amyloid precursor protein expression. Neurobiol. Dis..

[118] Iqbal K., Alonso A.D.C., Chohan M.O., El-Akkad E., Gong C., Khatoon S., Liu F., Grundke-Iqbal I. (2007). Molecular Basis of Tau Protein Pathology: Role of Abnormal Hyperphosphorylation.

[119] Mroczko B., Groblewska M., Litman-Zawadzka A., Kornhuber J., Lewczuk P. (2018). Cellular Receptors of Amyloid β Oligomers (AβOs) in Alzheimer’s Disease. Int. J. Mol. Sci..

[120] Wang X., Tan L., Lu Y., Peng J., Zhu Y., Zhang Y., Sun Z. (2015). MicroRNA-138 promotes tau phosphorylation by targeting retinoic acid receptor alpha. FEBS Lett..

[121] Banzhaf-Strathmann J., Benito E., May S., Arzberger T., Tahirovic S., Kretzschmar H., Fischer A., Edbauer D. (2014). MicroRNA-125b induces tau hyperphosphorylation and cognitive deficits in Alzheimer’s disease. EMBO J..

[122] Hébert S.S., Wang W.-X., Zhu Q., Nelson P.T. (2013). A Study of Small RNAs from Cerebral Neocortex of Pathology-Verified Alzheimer’s Disease, Dementia with Lewy Bodies, Hippocampal Sclerosis, Frontotemporal Lobar Dementia, and Non-Demented Human Controls. J. Alzheimer’s Dis..

[123] Smith P.Y., Hernandez-Rapp J., Jolivette F., Lecours C., Bisht K., Goupil C., Dorval V., Parsi S., Morin F., Planel E. (2015). MiR-132/212 deficiency impairs tau metabolism and promotes pathological aggregation in vivo. Hum. Mol. Genet..

[124] Yang G., Song Y., Zhou X., Deng Y., Liu T., Weng G., Yu D., Pan S. (2015). MicroRNA-29c targets β-site amyloid precursor protein-cleaving enzyme 1 and has a neuroprotective role in vitro and in vivo. Mol. Med. Rep..

[125] Zhang J., Hu M., Teng Z., Tang Y.-P., Chen C. (2014). Synaptic and Cognitive Improvements by Inhibition of 2-AG Metabolism Are through Upregulation of MicroRNA-188-3p in a Mouse Model of Alzheimer’s Disease. J. Neurosci..

[126] Lee K., Kim J.-H., Kwon O.-B., An K., Ryu J., Cho K., Suh Y.-H., Kim H.-S. (2012). An Activity-Regulated microRNA, miR-188, Controls Dendritic Plasticity and Synaptic Transmission by Downregulating Neuropilin-2. J. Neurosci..

[127] Vilardo E., Barbato C., Ciotti M., Cogoni C., Ruberti F. (2010). MicroRNA-101 regulates amyloid precursor protein expression in hippocampal neurons. J. Biol. Chem..

[128] Liu W., Liu C., Zhu J., Shu P., Yin B., Gong Y., Qiang B., Yuan J., Peng X. (2012). MicroRNA-16 targets amyloid precursor protein to potentially modulate Alzheimer’s-associated pathogenesis in SAMP8 mice. Neurobiol. Aging.

[129] Zhang B., Chen C.-F., Wang A.-H., Lin Q.-F. (2015). MiR-16 regulates cell death in Alzheimer’s disease by targeting amyloid precursor protein. Eur. Rev. Med. Pharmacol. Sci..

[130] Delay C., Calon F., Mathews P., Hébert S.S. (2011). Alzheimer-specific variants in the 3’UTR of Amyloid precursor protein affect microRNA function. Mol. Neurodegener..

[131] Hébert S.S., Papadopoulou A.S., Smith P., Galas M.C., Planel E., Silahtaroglu A.N., Sergeant N., Buée L., de Strooper B. (2010). Genetic ablation of dicer in adult forebrain neurons results in abnormal tau hyperphosphorylation and neurodegeneration. Hum. Mol. Genet..

[132] Zhu X., Yao Y., Liu Y., Zhou R., Zhang W., Hu Q., Liu H., Al Hamda M.H., Zhang A. (2019). Regulation of ADAM10 by MicroRNA-23a Contributes to Epileptogenesis in Pilocarpine-Induced Status Epilepticus Mice. Front. Cell Neurosci..

[133] Cheng C., Li W., Zhang Z., Yoshimura S., Hao Q., Zhang C., Wang Z. (2013). MicroRNA-144 is regulated by activator protein-1 (AP-1) and decreases expression of alzheimer disease-related a disintegrin and metalloprotease 10 (ADAM10). J. Biol. Chem..

[134] Kuhn P.H., Colombo A.V., Schusser B., Dreymueller D., Wetzel S., Schepers U., Herber J., Ludwig A., Kremmer E., Montag D. (2016). Systematic substrate identification indicates a central role for the metalloprotease ADAM10 in axon targeting and synapse function. Elife.

[135] Huang X., Yuan T., Tschannen M., Sun Z., Jacob H., Du M., Liang M., Dittmar R.L., Liu Y., Liang M. (2013). Characterization of human plasma-derived exosomal RNAs by deep sequencing. BMC Genomics..

[136] Alzheimer’s Disease International (2019). World Alzheimer Report 2019: Attitudes to Dementia.

[137] Landau S.M., Harvey D., Madison C.M., Reiman E.M., Foster N.L., Aisen P.S., Petersen R.C., Shaw L.M., Trojanowski J.Q., Jack C.R. (2010). Comparing predictors of conversion and decline in mild cognitive impairment. Neurology.

[138] Sperling R.A., Aisen P.S., Beckett L.A., Bennett D.A., Craft S., Fagan A.M., Iwatsubo T., Jack C.R., Kaye J., Montine T.J. (2011). Toward defining the preclinical stages of Alzheimer’s disease: Recommendations from the National Institute on Aging-Alzheimer’s Association workgroups on diagnostic guidelines for Alzheimer’s disease. Alzheimer’s Dement..

[139] Jack C.R., Knopman D.S., Jagust W.J., Petersen R.C., Weiner M.W., Aisen P.S., Shaw L.M., Vemuri P., Wiste H.J., Weigand S.D. (2013). Tracking pathophysiological processes in Alzheimer’s disease: An updated hypothetical model of dynamic biomarkers. Lancet Neurol..

[140] El Kadmiri N., Said N., Slassi I., El Moutawakil B., Nadifi S. (2018). Biomarkers for Alzheimer Disease: Classical and Novel Candidates’ Review. Neuroscience.

[141] Chételat G., Arbizu J., Barthel H., Garibotto V., Law I., Morbelli S., van de Giessen E., Agosta F., Barkhof F., Brooks D.J. (2020). Amyloid-PET and 18F-FDG-PET in the diagnostic investigation of Alzheimer’s disease and other dementias. Lancet Neurol..

[142] Ritchie C., Smailagic N., Noel-Storr A.H., Takwoingi Y., Flicker L., Mason S.E., Mcshane R. (2014). Plasma and cerebrospinal fluid amyloid beta for the diagnosis of Alzheimer’s disease dementia and other dementias in people with mild cognitive impairment (MCI). Cochrane Database Syst. Rev..

[143] Preische O., Schultz S.A., Apel A., Kuhle J., Kaeser S.A., Barro C., Gräber S., Kuder-Buletta E., LaFougere C., Laske C. (2019). Serum neurofilament dynamics predicts neurodegeneration and clinical progression in presymptomatic Alzheimer’s disease. Nat. Med..

[144] Khalil M., Teunissen C.E., Otto M., Piehl M., Sormani M.P., Gattringer T., Barro C., Kappos L., Comabella M., Fazekas F. (2018). Neurofilaments as biomarkers in neurological disorders. Nat. Rev. Neurol..

[145] Swarbrick S., Wragg N., Ghosh S., Stolzing A. (2019). Systematic Review of miRNA as Biomarkers in Alzheimer’s Disease. Mol. Neurobiol..

[146] Zhao Y., Jaber V., Alexandrov P.N., Vergallo A., Lista S., Hampel H., Lukiw W.J. (2020). microRNA-Based Biomarkers in Alzheimer’s Disease (AD). Front. Neurosci..

[147] Riancho J., Vázquez-Higuera J.L., Pozueta A., Lage C., Kazimierczak M., Bravo M., Calero M., Gonalezález A., Rodríguez E., Lleó A. (2017). MicroRNA Profile in Patients with Alzheimer’s Disease: Analysis of miR-9-5p and miR-598 in Raw and Exosome Enriched Cerebrospinal Fluid Samples. J. Alzheimer’s Dis..

[148] Geekiyanage H., Jicha G.A., Nelson P.T., Chan C. (2012). Blood serum miRNA: Non-invasive biomarkers for Alzheimer’s disease. Exp. Neurol..

[149] Barbagallo C., Mostile G., Baglieri G., Giunta F., Luca A., Raciti L., Zappia M., Purrello M., Ragusa M., Nicoletti A. (2020). Specific Signatures of Serum miRNAs as Potential Biomarkers to Discriminate Clinically Similar Neurodegenerative and Vascular-Related Diseases. Cell Mol. Neurobiol..

[150] Guo R., Fan G., Zhang J., Wu C., Du Y., Ye H., Li Z., Wang L., Zhang Z., Zhang L. (2017). A 9-microRNA Signature in Serum Serves as a Noninvasive Biomarker in Early Diagnosis of Alzheimer’s Disease. J. Alzheimer’s Dis..

[151] Denk J., Oberhauser F., Kornhuber J., Wiltfang J., Fassbender K., Schroeter M.L., Volk A.E., Diehl-Schmid J., Prudlo J., Danek A. (2018). Specific serum and CSF microRNA profiles distinguish sporadic behavioural variant of frontotemporal dementia compared with Alzheimer patients and cognitively healthy controls. PLoS ONE..

[152] Galimberti D., Villa C., Fenoglio C., Serpente M., Ghezzi L., Cioffi S.M.G., Arighi A., Fumagalli G., Scarpini E. (2014). Circulating miRNAs as Potential Biomarkers in Alzheimer’s Disease. J. Alzheimer’s Dis..

[153] Satoh J.I., Kino Y., Niida S. (2015). MicroRNA-Seq data analysis pipeline to identify blood biomarkers for alzheimer’s disease from public data. Biomark Insights.

[154] Kiko T., Nakagawa K., Tsuduki T., Furukawa K., Arai H., Miyazawa T. (2014). MicroRNAs in plasma and cerebrospinal fluid as potential markers for Alzheimer’s disease. J. Alzheimer’s Dis..

[155] Müller M., Jäkel L., Bruinsma I.B., Claassen J.A., Kuiperij H.B., Verbeek M.M. (2016). MicroRNA-29a Is a Candidate Biomarker for Alzheimer’s Disease in Cell-Free Cerebrospinal Fluid. Mol. Neurobiol..

[156] Cosín-Tomás M., Antonell A., Lladó A., Alcolea D., Fortea G., Ezquerra M., Lleó A., Martí M.J., Pallàs M., Sanchez-Valle R. (2017). Plasma miR-34a-5p and miR-545-3p as Early Biomarkers of Alzheimer’s Disease: Potential and Limitations. Mol. Neurobiol..

[157] Wang J., Chen C., Zhang Y. (2020). An investigation of microRNA-103 and microRNA-107 as potential blood-based biomarkers for disease risk and progression of Alzheimer’s disease. J. Clin. Lab. Anal..

[158] Tan L.L., Yu J.-T.T., Liu Q.-Y.Y., Tan M.-S.S., Zhang W., Hu N., Wang Y.-L.L., Sun L., Jiang T., Tan L.L. (2014). Circulating miR-125b as a biomarker of Alzheimer’s disease. J. Neurol. Sci..

[159] Sheinerman K.S., Tsivinsky V.G., Abdullah L., Crawford F., Umansky S.R. (2013). Plasma microRNA biomarkers for detection of mild cognitive impairment: Biomarker validation study. Aging.

[160] Cha D.J., Mengel D., Mustapic M., Liu W., Selkoe D.J., Kapogiannis D., Galasko D., Rissman R.A., Bennett D.A., Walsh D.M. (2019). miR-212 and miR-132 Are Downregulated in Neurally Derived Plasma Exosomes of Alzheimer’s Patients. Front. Neurosci..

[161] Xie B., Zhou H., Zhang R., Song M., Yu L., Wang L., Liu Z., Zhang Q., Cui D., Wang X. (2015). Serum miR-206 and miR-132 as Potential Circulating Biomarkers for Mild Cognitive Impairment. J. Alzheimer’s Dis..

[162] Ansari A., Maffioletti E., Milanesi E., Marizzoni M., Frisoni G.B., Blin O., Richardson J.C., Bordet R., Forloni G., Gennarelli M. (2019). miR-146a and miR-181a are involved in the progression of mild cognitive impairment to Alzheimer’s disease. Neurobiol. Aging.

[163] Siedlecki-Wullich D., Català-Solsona J., Fábregas C., Hernández I., Clarimon J., Lleó A., Boada M., Saura C.A., Rodríguez-Álvarez J., Miñano-Molina A.J. (2019). Altered microRNAs related to synaptic function as potential plasma biomarkers for Alzheimer’s disease. Alzheimer’s Res. Ther..

[164] Kumar P., Dezso Z., MacKenzie C., Oestreicher J., Agoulnik S., Byrne M., Bernier F., Yanagimachi M., Aoshima K., Oda Y. (2013). Circulating miRNA biomarkers for Alzheimer’s disease. PLoS ONE.

[165] Kenny A., McArdle H., Calero M., Rabano A., Madden S.F., Adamson K., Forster R., Spain E., Prehn J.H.M., Henshall D.C. (2019). Elevated plasma microRNA-206 levels predict cognitive decline and progression to dementia from mild cognitive impairment. Biomolecules.

[166] Gámez-Valero A., Campdelacreu J., Vilas D., Ispierto L., Reñé R., Álvarez R., Armengol M.P., Borràs F.E., Beyer K. (2019). Exploratory study on microRNA profiles from plasma-derived extracellular vesicles in Alzheimer’s disease and dementia with Lewy bodies. Transl. Neurodegener..

[167] Yu L., Li H., Liu W., Zhang L., Tian Q., Li H., Li M. (2020). MiR-485-3p serves as a biomarker and therapeutic target of Alzheimer’s disease via regulating neuronal cell viability and neuroinflammation by targeting AKT3. Mol. Genet. Genomic Med..

[168] Hara N., Kikuchi M., Miyashita A., Hatsuta H., Saito Y., Kasuga K., Murayama S., Ikeuchi T., Kuwano R. (2017). Serum microRNA miR-501-3p as a potential biomarker related to the progression of Alzheimer’s disease. Acta Neuropathol. Commun..

[169] Hajian-Tilaki K. (2013). Receiver Operating Characteristic (ROC) Curve Analysis for Medical Diagnostic Test Evaluation. Casp. J. Intern. Med..

[170] Sheinerman K.S., Toledo J.B., Tsivinsky V.G., Irwin D., Grossman M., Weintraub D., Hurtig H.I., Chen-Plotkin A., Wolk D.A., McCluskey L.F. (2017). Circulating brain-enriched microRNAs as novel biomarkers for detection and differentiation of neurodegenerative diseases. Alzheimers Res. Ther..

[171] Leidinger P., Backes C., Deutscher S., Schmitt K., Mueller S.C., Frese K., Haas J., Ruprecht K., Paul F., Stähler C. (2013). A blood based 12-miRNA signature of Alzheimer disease patients. Genome Biol..

[172] Jain G., Stuendl A., Rao P., Berulava T., Pena Centeno T., Kaurani L., Burkhardt S., Delalle I., Kornhuber J., Hüll M. (2019). A combined miRNA–piRNA signature to detect Alzheimer’s disease. Transl. Psychiatry.

[173] Souza V.C., Morais G.S., Henriques A.D., Machado-Silva W., Perez D.I.V., Brito C.J., Camargos E.F., Moraes C.F., Nóbrega O.T. (2020). Whole-Blood Levels of MicroRNA-9 Are Decreased in Patients With Late-Onset Alzheimer Disease. Am. J. Alzheimer’s Dis. Other Dementiasr..

[174] Denk J., Boelmans K., Siegismund C., Lassner D., Arlt S., Jahn H. (2015). MicroRNA Profiling of CSF Reveals Potential Biomarkers to Detect Alzheimer`s Disease. Hoheisel JD, ed. PLoS ONE.

[175] Lugli G., Cohen A.M., Bennett D.A., Shah R.C., Fields C.J., Hernandez A.G., Smalheiser N.R. (2015). Plasma Exosomal miRNAs in Persons with and without Alzheimer Disease: Altered Expression and Prospects for Biomarkers. PLoS ONE.

[176] Valadi H., Ekström K., Bossios A., Sjöstrand M., Lee J.J., Lötvall J.O. (2007). Exosome-mediated transfer of mRNAs and microRNAs is a novel mechanism of genetic exchange between cells. Nat. Cell Biol..

[177] Chevillet J.R., Kang Q., Ruf I.K., Briggs H.A., Vojtech L.N., Hughes S.M., Cheng H.H., Arroyo J.D., Meredith E.K., Gallichotte E.N. (2014). Quantitative and stoichiometric analysis of the microRNA content of exosomes. Proc. Natl. Acad. Sci. USA.

[178] Endzelinš E., Berger A., Melne V., Bajo-Santos C., Sobolevska K., Abols A., Rodriguez M., Šantare D., Rudnickiha A., Lietuvietis V. (2017). Detection of circulating miRNAs: Comparative analysis of extracellular vesicle-incorporated miRNAs and cell-free miRNAs in whole plasma of prostate cancer patients. BMC Cancer.

[179] Angelucci F., Cechova K., Valis M., Kuca K., Zhang B., Hort J. (2019). MicroRNAs in Alzheimer’s Disease: Diagnostic Markers or Therapeutic Agents?. Front. Pharmacol..

[180] Gabr M.T., Brogi S. (2020). MicroRNA-Based Multitarget Approach for Alzheimer’s Disease: Discovery of the First-In-Class Dual Inhibitor of Acetylcholinesterase and MicroRNA-15b Biogenesis. J. Med Chem..

[181] Jahangard Y., Monfared H., Moradi A., Zare M., Mirnajafi-Zadeh J., Mowla S.J. (2020). Therapeutic Effects of Transplanted Exosomes Containing miR-29b to a Rat Model of Alzheimer’s Disease. Front. Neurosci..

